# Ciliary flow and morphology shape mass transport at the surface and within gastrovascular cavities of black corals

**DOI:** 10.1038/s42003-026-10531-2

**Published:** 2026-06-30

**Authors:** Mathilde Godefroid, Francisco Otero-Ferrer, Michael Wind-Hansen, Lars Behrendt, Ignace Ransquin, Emilio Soler-Onís, Fernando Espino, Sten Littmann, Soeren Ahmerkamp

**Affiliations:** 1 Department of Biogeochemistry, Max Planck Institute for Marine Microbiology, Bremen, Germany; 2 Department of Organismal Biology, Group of Ecological and Evolutionary Genomics, Université Libre de Bruxelles, Brussels, Belgium; 3 Biodiversity & Conservation Research Group, IU-ECOAQUA, Scientific & Technological Marine Park of the Universidad de Las Palmas de Gran Canaria, Telde, Spain; 4 Department of Biology – Section for Microbiology, Aarhus University, Aarhus, Denmark; 5 Science for Life Laboratory, Department of Organismal Biology, Program of Environmental Toxicology, Uppsala University, Uppsala, Sweden; 6 Department of Biotechnology and Biomedicine, Section for Microbial and Chemical Ecology, Technical University of Denmark, Lyngby, Denmark; 7 Institute of Physics, School of Mathematics and Science, Carl von Ossietzky Universität Oldenburg, University of Oldenburg, Oldenburg, Germany; 8 Biological Oceanography Section, Leibniz Institute for Baltic Sea Research Warnemünde (IOW), Rostock, Germany

**Keywords:** Ecophysiology, Biophysics, Optical imaging, Respiration

## Abstract

Sessile marine organisms rely on morphology to mediate interactions with their environment, yet the microscale drivers of morphological diversification remain incompletely resolved. Black corals, with their high morphological repertoire and global ecological importance, provide an ideal taxon to explore these drivers. Using high-speed videography, microscale particle image velocimetry, oxygen-sensitive sensPIV, and microsensors, we quantified how colony morphology influences hydrodynamics on polyp to colony scales, and how these flow environments regulate mass transport and exchange at tissue surfaces. We document beating epidermal cilia in black corals and reveal that species deploy distinct morphology-cilia combinations to meet metabolic needs, revealing a trade-off between enhancing exchange surface area and maintaining effective ventilation. By visualizing cilia-driven circulation within gastrovascular cavities, we demonstrate strong internal connectivity shaped by skeletal spines, again revealing how morphology governs ciliary function. This form-function feedback highlights ciliary dynamics as a key but previously overlooked driver of performance, habitat specialization, and diversification in sessile life.

## Abstract (web summary)

Mechanistic analysis of morphology–cilia interactions in black corals reveals how hydrodynamics and ciliary transport shape metabolic exchange, internal connectivity, and morphological diversification in sessile marine organisms.

## Introduction

A remarkable diversity of sessile organisms thrives on the seafloor, exposed directly to the physicochemical conditions of their environment, with no possibility to escape. These organisms live at risk of being displaced by currents, but also depend on their environment to acquire nutrients, remove waste products and disperse their larvae^1^. Sessile organisms therefore, exhibit a range of strategies to cope with local flow regimes. In low-flow environments, they may generate feeding currents or tolerate reduced oxygen levels, while those in high-flow environments develop morphologies that increase resistance and resource acquisition^1^.

Among sessile organisms, reef-building corals received particular attention, notably because coral reefs are among the most diverse ecosystems on the planet^2^, supporting multiple ecosystem services^3^, but also because they are increasingly threatened by climate change. Flow plays a key role in coral physiology by influencing mass transfer and thermal stress. For instance, increased flow can mitigate coral bleaching by enhancing heat and solute exchange^4,5^. This has motivated extensive work on the relationship between flow and coral morphology^6,7^. In high-flow environments, branching corals develop more compact morphologies, whereas low-flow environments select for thinner, elongated morphologies^8^. These adaptations themselves impose hydrodynamic changes within and around colonies^9–11^, creating a feedback loop that influences organismal resistance, energy acquisition and feeding, nutrient uptake, waste removal and gas exchange^12–14^.

At the colony scale (cm-m), flow enhances mass transfer through advection and turbulence^15,16^, and is further modulated by colony morphology, with branching density and surface topography generating complex flow fields, including passive vortices within colony interiors^11,17,18^. As flow approaches the tissue surface, it is progressively attenuated, forming a thin flow boundary layer. Within this sub-millimeter region, below the Kolmogorov microscale (η ≈ 10⁻³ m in seawater; e.g., ref. ^19^), turbulence is dissipated and viscous forces dominate. Here, mass-transfer is dominated by molecular diffusion, and the resulting slow transport leads to the formation of a diffusive boundary layer (DBL), which thickness governs the exchange of oxygen, nutrients and waste products^20^. For corals, the DBL thickness, operationally defined as the point where solute concentrations reach ~99%, depends on ambient flow^12,21–23^, is influenced by surface topography and polyp behavior, creating a coral-specific boundary layer, hereafter referred to as “coral boundary layer” (CBL)^24–29^.

Scleractinian corals were shown to actively overcome diffusion-limitations within the CBL by using cilia^30^. Cilia are highly conserved hair-like structures that emerged a billion years ago in the first eukaryotic cells^31^. In scleractinian corals, cilia stir the CBL and create vortices that enhance mass transfer by up to 400-fold compared to diffusion alone^30^. Ciliary flows were associated with feeding^32–36^, cleansing^37–41^, pathogen removal^42–47^, and symbiont recruitment^48^, and have been shown to enhance oxygen transport^49^, mitigate oxidative stress^50,51^ and increase within-colony exchanges by connecting neighboring polyps at the tissue surface^52^. In addition, ciliary flow also occurs in the interior of coral colonies. Here, individual polyps are connected via gastrovascular canals lined with ciliated cells that create internal fluid flows^53^. Models of the internal ciliary flow suggest that these enhance overall mass transfer processes by effectively extending the surface area of a coral^52^.

Coral morphology creates hydrodynamic microenvironments, while ciliary activity locally enhances transport and exchange, however, ciliary function itself depends on the flow conditions imposed by coral morphology, coupling both. Despite this, the interdependence of coral morphology and ciliary function remains largely unexplored. To investigate this reciprocal coupling, antipatharians (Cnidaria: Hexacorallia), or black corals, represent a promising taxon due to their exceptional morphological diversity. These non-photosynthetic corals inhabit environments from shallow waters to the deep sea^54^. They function as ecosystem-engineers by creating three-dimensional structures over the seafloor and attracting numerous associated species. Their colony morphologies range from whip-like to complex branching patterns, yet what determines their morphological diversity remains poorly understood. Currents generated by ciliary activity have been documented in three antipatharian species, suggesting a role in feeding^36^. Studies reported morphological differences among black corals that correlate with variation in biomechanical properties, feeding strategies, sediment load and heat-stress tolerance^55–57^. For instance, whip-like species can maintain their axis vertical, whereas branched species can bend over, suggesting contrasting feeding strategies associated with specific biomechanical properties of the stem^55^. However, branched morphologies also appear to be more vulnerable to thermal stress, possibly due to diffusion limitations within the colony^56^. Evolutionary analyses suggested that morphological adaptations of black corals have influenced their invasion and persistence in different habitats and depths through time^58^. Given their ecological and evolutionary importance, understanding the mechanisms governing black coral morphology offers more than taxon-specific insight: it positions black corals as a model system for uncovering the general principles that link form and function, to the ecology and evolution of sessile life in the ocean.

In this study, we investigate how differences in black coral morphology influence mass transport through (1) morphology-related hydrodynamic microenvironments and (2) the functional role of cilia. We focus on two co-occurring species with contrasting morphologies but high local abundance. We combine multi-scale flow visualization, using microscale particle image and tracking velocimetry (µPIV, µPTV), and metabolic activity measurements, using oxygen-sensitive sensPIV^51^, microsensors and respirometry assays, to determine how morphology interacts with flow and physiology to shape ecological performance.

## Results and discussion

### Coral morphology influences flow patterns

**Fig. 1:**
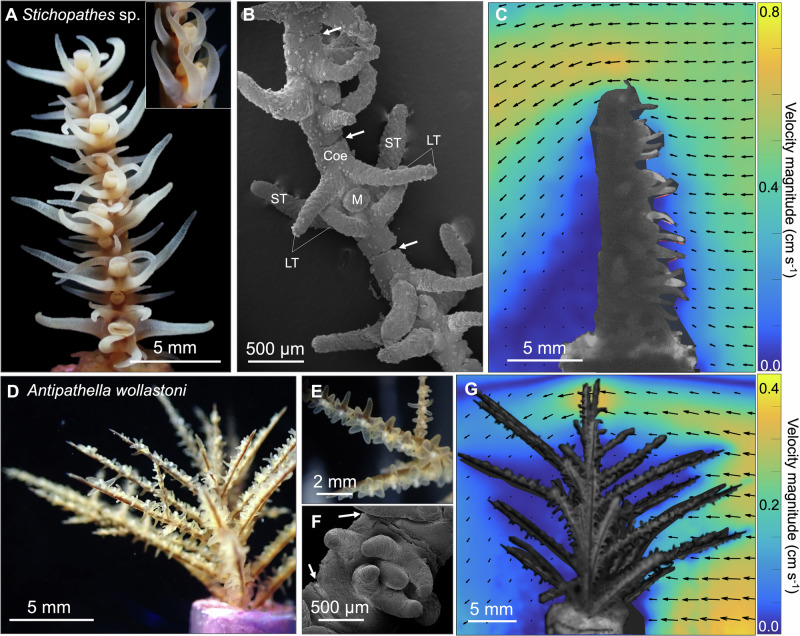
Morphological description and flow field analysis of fragments of *Stichopathes* sp. and *Antipathella wollastoni.* **A**, **D** Photograph of the fragment (Inset in **A**: contracted polyps of *Stichopathes* sp.). Surface area-to-volume ratio is 6.3 ± 0.4 and 17.6 ±﻿ 0.7 (mean ± SE) for *Stichopathes* sp. and *A. wollastoni*, respectively (Fig. S1). **E** Close-up on the polyps of *A. wollastoni*. **B**, **F** Scanning electron microscopy of the polyps. M mouth, ST sagittal tentacles, LT lateral tentacles White arrows: gastrovascular cavity separations in the coenosarc (Coe) between adjacent polyps. **C**, **G** Flow fields around the fragments. Black arrows: flow direction and magnitude. Color scale: flow velocity magnitude (cm s^−1^), calculated using PIVlab (MATLAB toolbox).

We studied two black coral species with contrasting morphologies (Fig. 1). *Stichopathes* sp. is a wire-like, unbranched species, whereas *Antipathella wollastoni* is bushy with dense branching. Both species harbor polyps on only one side of their main axis, distinguishing a polyp-bearing and polyp-free side. Polyp size differs markedly: *Stichopathes* sp. has ~14x larger polyps with higher intra-colonial size variability (39.7 ± 3.5 mm^2^, mean ± SE) than *A. wollastoni* (2.9 ± 0.0 mm^2^) (Table S1). Being hexacorals, polyps bear six tentacles surrounding the mouth. In *Stichopathes* sp., the two sagittal tentacles are ~1.5x longer than the four lateral ones, suggesting functional differentiation, whereas tentacles in *A. wollastoni* are uniform in size.

We investigated how seawater flow interacts with *Stichopathes* sp. and *A. wollastoni* using particle image velocimetry (PIV) at the scale of individual fragments (~3 cm) (Fig. 1C, G). Flow conditions encountered at 50 m depth near the collection sites typically vary between 1 cm s^−1^ (10th percentile) and 26 cm s^−1^ (90th percentile), with a median of ~7 cm s^−1^^59^. The imposed flow conditions (between approx. 0.4–1.0 cm s^−1^) are at the lower end of in situ free-stream velocity but account for the rapid decay of flow within the benthic boundary layer toward the viscous sublayer^60^. Comparable flow regimes have also been applied in previous studies investigating particle capture and colony fitness on *A. wollastoni*^59,61^ and on scleractinian corals^49,51^. In *Stichopathes* sp., flow (~0.4 cm s^−1^) directed towards the polyp-bearing side accelerated at the tip of the fragment and was reduced by ~91% on the sheltered side (i.e., lee side) (Fig. 1C). The stem diameter of *Stichopathes* sp. was 1.5 ± 0.4 mm (mean ± SD; *n* = 7) (Fig. S1), indicating a Reynolds number of 5.9. In *A. wollastoni*, flow (~0.4 cm s^−1^) penetrated the open, branched structure but was reduced by 89% inside the colony relative to the exterior (Fig. 1G). Branch diameter was 0.45 ± 0.1 (*n* = 7) (Fig. S1), indicating a Reynolds number of 1.8. This attenuation in flow velocity persisted under elevated flow conditions (~1 cm s^−1^), indicating a consistent feature in branched corals. Flow patterns were consistent across replicates for both species. This shows that, in addition to flow reduction on the lee side, branched colony morphologies also attenuate flow within the branches, a pattern not observed in whip-like morphologies.

### Species-specific ciliary flow

**Fig. 2:**
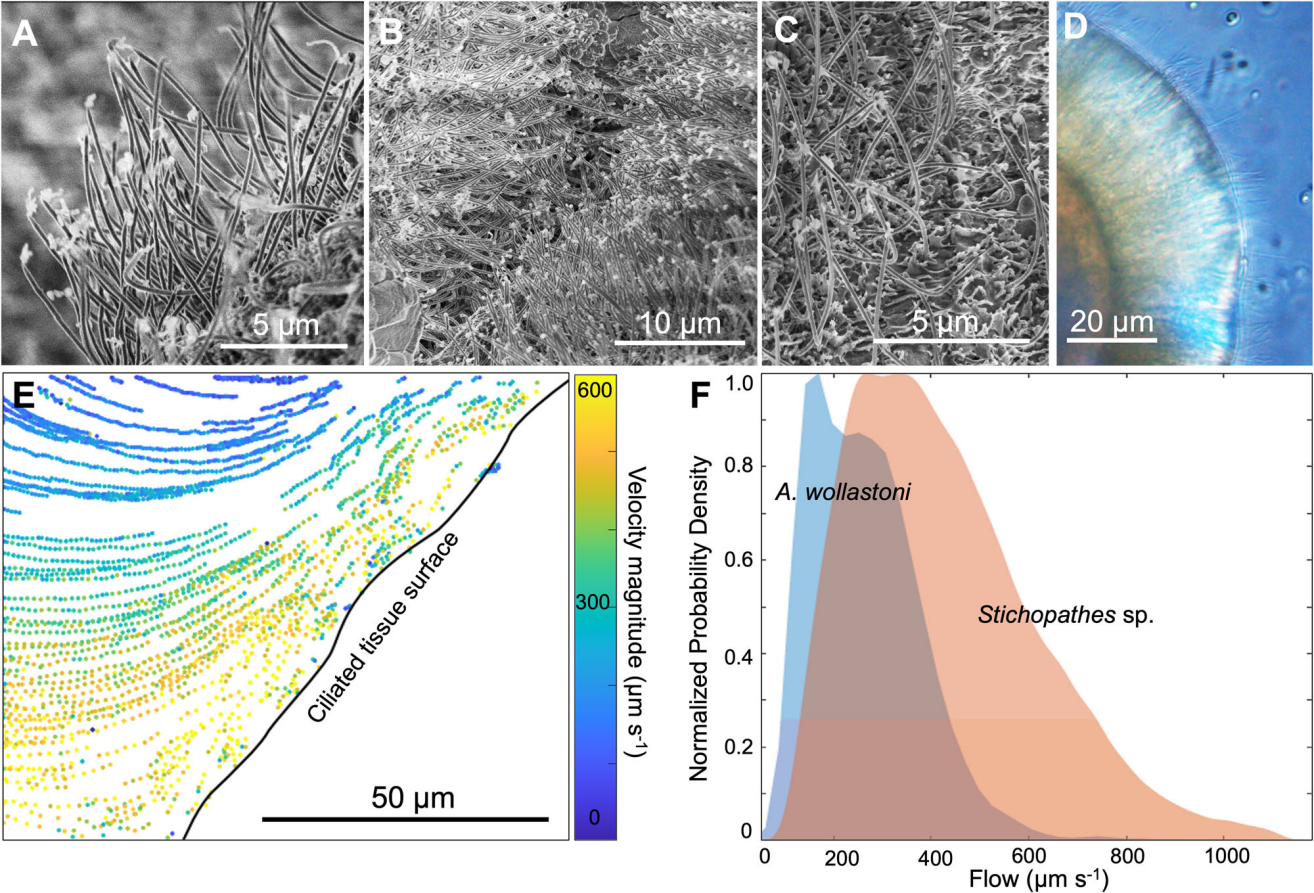
Antipatharian ciliary sheets and microscale flow velocities. SEM secondary electron micrograph images at the surface of the tentacle (**A**), the mouth opening (**B**), and the coenosarc (**C**) of *Antipathella wollastoni*. **D** Light microscopy image of cilia at the tentacle surface of *Stichopathes* sp. **E** Velocity flow field in the immediate vicinity of the surface of *A. wollastoni*, showing highest velocities near the boundary, driven by ciliary activity (Movie S2). **F** Comparison of velocity magnitude distributions close to the tissue surface of *Stichopathes* sp. and *A. wollastoni* (in red and blue, respectively).

On sub-millimeter scales, the interaction between corals and their environment is shaped by the activity of cilia at the tissue surface^30^. However, beyond early reports suggesting a function in generating feeding currents^35^, the role of cilia in antipatharians has not been systematically investigated. Using microscopy techniques (Fig. 2A–D) we identified cilia on the tentacles, the coenosarc, and on the mouth wall and interior of both species. Cilia length was nearly identical between *Stichopathes* sp. (10.6 ± 0.7 µm, *n* = 4) and *A. wollastoni* (10.4 ± 0.2 µm, *n* = 20). Image analysis revealed that average cilia density was ~13 × 10^6^ cilia cm^−2^ in *A. wollastoni* (*n* = 16) and ~11 × 10^6^ cilia cm^−2^ in *Stichopathes* sp. (*n* = 6) (all locations pooled), approximately fourfold higher than scleractinian corals^30^. Cilia density did not significantly differ across locations (tentacle, mouth edge and coenosarc) or species (two-way ANOVA, *p* > 0.5).

To resolve how ciliary motion influences local flow dynamics, we used high-speed videography capable of capturing the stroke cycles of individual cilia (Movie S1). In *A. wollastoni*, cilia beat at frequencies of 15–18 strokes s^−1^, while in *Stichopathes* sp., frequencies were 10–13 strokes s^−1^ (Fig. S2). These values fall within the range reported for other aquatic invertebrates, where ciliary stroke frequencies typically span 10–30 Hz depending on species, environmental conditions, and physiological state^62^. The slightly reduced frequencies in *Stichopathes* sp. may reflect differences in colony architecture, tissue density, or metabolic demand compared to *A. wollastoni*. By tracking the displacement of small tracer particles along the ciliated tissue surface using particle tracking velocimetry (PTV), we identified well-organized flow structures, with pronounced particle acceleration near the ciliated surfaces (Fig. 2E, Movie S2). Average flow velocities reached 271 ± 3 µm s^−1^ in *A. wollastoni*, with peaks up to 409 µm s^−1^. In *Stichopathes* sp., average near-surface velocities were 341 ± 3 µm s^−1^, with the 90th percentile approaching 541 µm s^−1^ (Fig. 2F). This flow topology is characteristic of densely packed, metachronally beating cilia and mirrors the propulsion patterns observed in mucociliary surfaces of scleractinian corals and bivalves, as well as in cilia used for locomotion in protists^63,64^. These values align with ciliary transport rates reported in other invertebrates, such as gastropod veligers that transport particles along food grooves at ~90–130 µm s^−1^^65^. However, flow velocities generated by metachronal waves across different organisms span more than three orders of magnitude (10^1^–10^4^ µm s^−1^), a trend attributed to variation in ciliary density and beating frequency^62^. Here, cilia length and density were comparable between species, but cilia beating frequency and flow speed generation were not correlated. Despite having a lower beating frequency, *Stichopathes* sp. generated faster flow than *A. wollastoni*. This mismatch between frequency and flow speed is likely driven by differences in the coordination of metachronal waves, i.e., how cilia are phase-locked across the tissue, which can substantially alter the efficiency of momentum transfer. Such coordination differences may reflect species-specific trade-offs between food capture and ventilation, motivating our examination of the resulting large-scale flow fields.

### Microscale morphology and ciliary dynamics generate vortices that enhance mass transfer

**Fig. 3:**
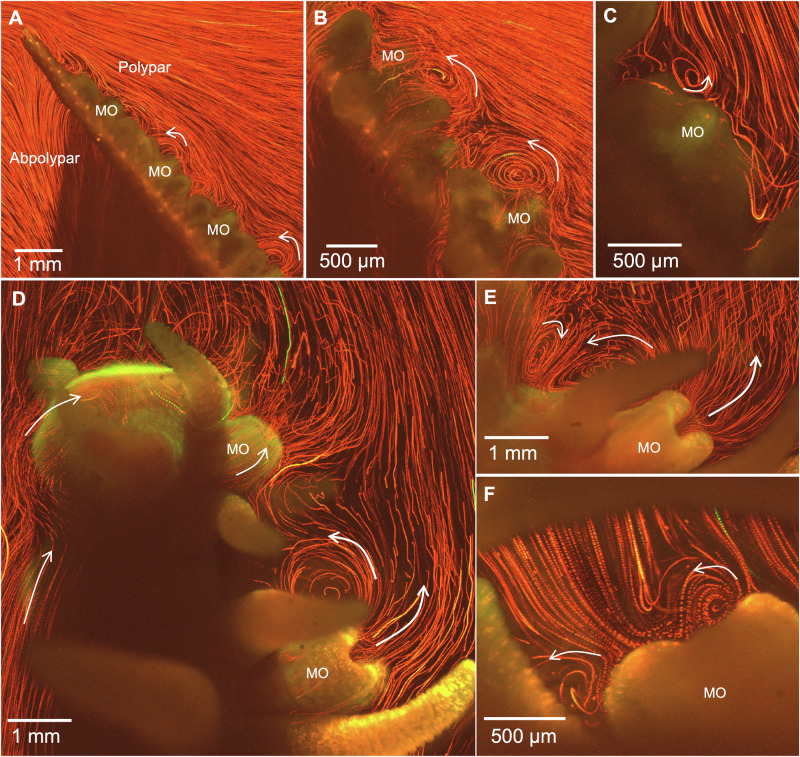
Ciliary flows interact with local coral topography to form ciliary vortices. **A**–**C** *Antipathella wollastoni* and **D**–**F** *Stichopathes* sp. White arrows depict flow direction. MO, Mouth Opening. Distinction between polyp-bearing and polyp-free sides of the stem are shown in (**A**). Note the upward-directed flows on the polyp-free side and mouth walls in **D**, the outward-directed flow at the mouth opening in **D**, **E**, the adjacent counter-rotating vortices in **E**, and the smaller vortices at the mouth opening in (**F**). Images were obtained by computing maximum-intensity projection of image stacks.

To examine how microenvironmental and morphological constraints influence interspecies differences in ciliary flow, we employed microscale PIV (µPIV) and image analysis (Maximum Intensity Projection), to directly visualize and characterize the cilia-induced vortices in both species (Fig. 3). The formation of large vortices was typically observed on the polyp-bearing side of the stem. Ciliary flow typically moves up towards the mouth opening and then extends laterally towards the tentacles (Fig. 3A, B). In some instances, this flow created counter-rotating vortices between two neighboring polyps (Fig. 3E). Finally, while no ciliary vortices were observed on the polyp-free side of any species, strong upward flow was present, carrying particles toward the apical tips (Fig. 3D). Ciliary flow was observed carrying particles toward the mouth openings, likely enhancing feeding efficiency or ventilation; in addition, particles were occasionally observed being ejected from the mouth (Movie S3), indicating that ciliary transport can also facilitate outward fluxes, such as waste removal.

Comparable vortical flows were observed on at least five other scleractinian coral species^64^, showing that these stirring flows are a widespread characteristic in hexacorals with beating epidermal cilia. Vortical ciliary flows differed in size between species. *Stichopathes* sp. produced large vortices (0.53 ± 0.06 mm, *n* = 11) between the mouth and neighboring polyps and smaller ones (0.25 ± 0.04 mm, *n* = 6) at the mouth opening when open, likely driven by pharyngeal cilia, whereas *A. wollastoni* generated only small vortices (0.28 ± 0.02 mm, *n* = 26) (Fig. 3F). These differences align with their polyp surface-to-volume ratios, with *Stichopathes* sp. exhibiting 2.8 times larger polyps and mouth openings than *A. wollastoni*. Together, these findings highlight the role of polyp size in shaping vortex size.

To quantify the increase in mass transfer provided by vortical ciliary flow in antipatharians, we calculated the Péclet number, which expresses the ratio of advective to diffusive transport rates over a given spatial scale. We obtained ${Pe}$ = 75–300, which is » 1, indicating a clear dominance of advection over diffusion in vortices. These values are in the same range as calculated in Ahmerkamp et al.^51^ (${Pe}$ = ~95), and in the lower range compared to scleractinian corals (${Pe}$ = 500–6000^25^). These data suggest that vortices generated by the activity of cilia considerably enhance mass transport to and from the tissue surface in antipatharian corals. This further supports the overall benefit of epidermal ciliary beating for solute exchange in hexacorals as a group. The degree to which this mechanism improves overall metabolic performance in black corals depends on how effectively ciliary activity alleviates substrate limitation at the tissue scale.

### Morphologies constrain oxygen exchange efficiency at colony scale

**Fig. 4:**
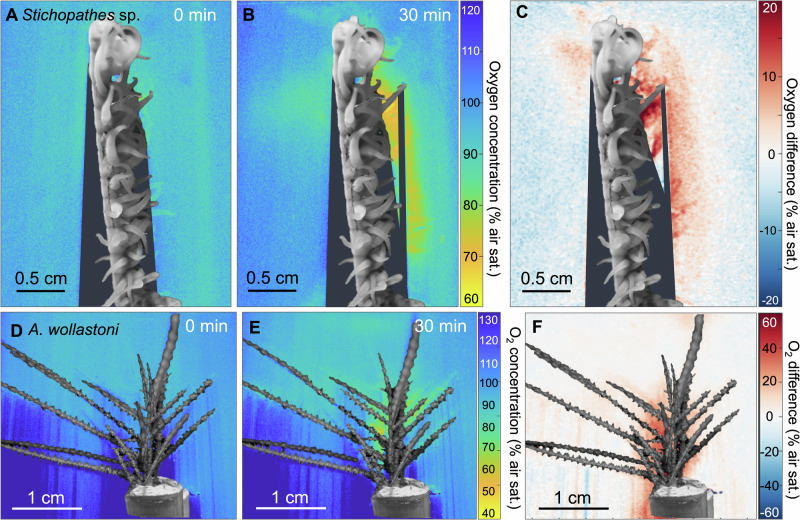
Oxygen concentration distribution around fragments of *Stichopathes* sp. and *Antipathella wollastoni.* **A**, **B**, **D**, **E** Spatial oxygen distribution (obtained via sensPIV) after 0 and 30 min in the absence of flow and in the dark. The dark blue stripes visible in *A. wollastoni* show areas shaded by the fragment in the laser sheet. Figure S3 shows all the time points until 60 min. **C**, **F** Oxygen concentration difference after 30 min in the absence of flow and in the dark. Areas of oxygen consumption are identified in red, by subtracting sensPIV images obtained after 30 min in the absence of flow (**B**, **E**) to images with low flow (**A**, **D**).

Oxygen-sensitive sensPIV offers a powerful method to directly visualize the oxygen distribution under flow in two dimensions^51,66^. We first applied this technique at fragment scale in both species of black corals in the absence of flow. This allowed us to investigate where low-oxygen regions develop over time and whether ciliary flow is sufficient to ventilate these regions and prevent hypoxia. When flow became quiescent, oxygen concentrations declined due to coral respiration, revealing spatial heterogeneities in oxygen distribution (Fig. 4 and Fig. S3). In unbranched *Stichopathes* sp., reduced oxygen concentrations were mostly observed on the polyp-bearing side of the stem, corresponding to the most active part of the fragment. In contrast, reduced oxygen concentrations were spatially confined to the colony interior in the branched *A. wollastoni*. These patterns were consistent across technical replicates in both species (Fig. S4) and likely reflect differences in metabolic activity or local mass transfer, including microscale transport enhancement by ciliary flow.

In situ, the formation of low-oxygen regions on the surface of corals will depend on local hydrodynamics. Well-ventilated regions exposed to incoming currents or located at the outer edges of a branched fragment are unlikely to experience oxygen limitation, as fully oxygenated seawater is continuously supplied. For unbranched *Stichopathes* sp., the extent of oxygen limitation may be governed by the orientation of the colony relative to the flow field: polyps facing the incoming current benefit from direct ventilation, whereas those on the sheltered side may experience reduced oxygen supply. In the branched *A. wollastoni*, low-oxygen areas were consistently associated with the central regions of the colony, where flow reduction is most pronounced, indicating that internal ventilation is limited even with active ciliary flow. While this study focused on oxygen, similar limitations likely apply to other molecules, suggesting that exchange between the colony interior and external seawater is generally limited in branched colonies.

### Ciliary flow increases net fluxes in *Stichopathes* sp. and transport of particles and solutes in *A. wollastoni*

**Fig. 5:**
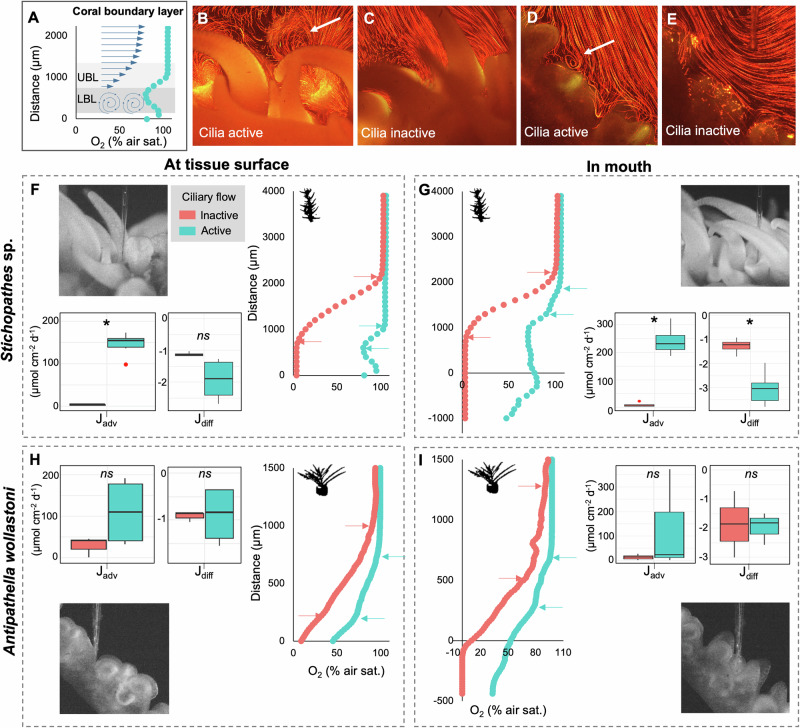
Oxygen concentration profiles and flux calculations across location and species, in the dark, with active (green) and inhibited (red) ciliary flow. **A** Typical S-shape profile with active ciliary flow, showing linear diffusion in the upper coral boundary layer and stirred conditions in the lower coral boundary layer. Blue arrows represent theoretical flow conditions. Maximum Intensity projections at the location where the microsensor measurements were taken, with active and inactive cilia, for *Stichopathes* sp. (**B**, **C**) and *A. wollastoni* (**D**, **E**). **F**–**I** Oxygen concentrations along a perpendicular transect from the tissue surface or the bottom of the mouth. Inserted boxplots show the advective (*J*_adv_) and diffusive (*J*_diff_) fluxes of oxygen. Arrows indicate limits of the upper CBL considered for diffusive flux calculations. Lower CBL was used to calculate advective flux (from tissue surface to first arrow along the profile). All profiles used in flux calculations are showed in Fig. S5. Source data underlying graph can be obtained from Supplementary Data. Statistical comparisons to test for ciliary flow effect using t-tests (Table S2). *ns*: not significant, *: significant (*p* < 0.05). Boxes represent the interquartile range (IQR) with the median (horizontal black line). Error bars represent the smallest and largest values within 1.5 x IQR and red dots represent outliers (>1.5 x IQR). Inset photographs show the location of the microsensor at the start of the profile.

To quantify oxygen transport driven by ciliary activity, we performed additional oxygen microsensor profiles at the tissue surface and in the mouth opening of both coral species under flow conditions (free stream velocities of approx. 0.4 cm s^−1^), providing microscale detail that complements the oxygen distribution obtained via sensPIV at the fragment-scale. Microsensor measurements commenced at the tissue surface or at the bottom of the mouth interior, and stopped above the CBL, where oxygen concentrations are constant (Fig. 5F–I). Measurements were performed with actively beating cilia and inhibited cilia, by addition of sodium orthovanadate, a ciliary inhibitor. PIV was used to verify ciliary inhibition upon addition of sodium orthovanadate (Fig. 5B–E).

The shape of oxygen profiles was strongly influenced by ciliary activity. This was particularly striking for *Stichopathes* sp., where S-shaped profiles were observed under actively beating cilia. Here, the oxygen concentration close to the tissue surface was reduced by 40 ± 5% air sat. (mean ± SE, *n* = 5) due to coral respiration, then rapidly increased in the CBL thanks to cilia moving oxygenated seawater into oxygen depleted regions. Upon addition of the ciliary inhibitor, the profile shape became linear, indicating mostly diffusive transport at the tissue surface. Under conditions with inhibited cilia, oxygen close to the tissue surface was depleted by 67 ± 4% air sat. (mean ± SE, *n* = 2) compared to active cilia, for *Stichopathes* sp. (Fig. 5F, G). In *A. wollastoni*, the effect of ciliary flow on profile shape was less striking (absence of S-shape; Fig. 5H, I). This is likely due to differences in polyp size between species, the smaller polyps of *A. wollastoni* providing less sheltering of the tissue surface from external flow conditions, compared to *Stichopathes* sp. This was evident from the specific differences in CBL thickness, being on average 3.4-fold greater in *Stichopathes* sp. (1837 ± 103 µm, *n* = 20) than in *A. wollastoni* (542 ± 65 µm, *n* = 20). Similarly, the average reduction in oxygen concentration upon ciliary inhibition was much less in *A. wollastoni* (21 ± 7% air sat., *n* = 3) compared to *Stichopathes* sp.

Further, we calculated diffusive flux in the upper CBL (above the close ciliary vortex) and advective flux in the lower CBL, where cilia are actively stirring (insets in Fig. 5F–I). Net diffusive fluxes towards coral tissue ranged from −0.25 to −4.95 µmol cm^−2^ d^−1^ in both species, with negative values indicating oxygen uptake. These net fluxes should be regarded as conservative estimates, as the three-dimensional nature of the coral morphology may introduce out-of-plane components, and additional unsteady dynamics, such as polyp movements, could further influence the net fluxes.

Ciliary activity had a particularly pronounced effect at the mouth opening of *Stichopathes* sp., where oxygen uptake increased significantly under active compared to inactive ciliary beating (t-test, *p* = 0.003, Table S2), highlighting the role of ciliary flow in respiration for this species. In contrast, advective fluxes were higher for both species (0 to 372 µmol cm^−2^ d^−1^) and were significantly enhanced under active compared to inactive cilia in *Stichopathes* sp. (*p* = 0.0004 in the mouth and *p* = 0.046 at the tissue surface), but not in *A. wollastoni*. Together, these results show that polyp size shapes CBL thickness and thus reliance on ciliary flow, with large-polyped *Stichopathes* sp. showing strong ciliary enhancement of advective flux and small-polyped *A. wollastoni* showing no significant effect. To assess the enhancement of coral-seawater exchanges by ciliary-induced flow relative to pure diffusion, we calculated the Sherwood number (Sh). Sh is a dimensionless number which expresses the ratio of advective to diffusive flux, based on fluxes calculated on the oxygen concentration profiles (For Sh calculations and discussion on limitations, see Supplementary Methods, Eq. 6–8). Measured Sh values were 78 and 48 for *Stichopathes* sp. and *A. wollastoni*, respectively, indicating that advective transport processes strongly dominate over diffusion in both species. A higher Sh denotes greater enhancement of exchange by advection, consistent with a more efficient ciliary-driven transport in *Stichopathes* sp. compared to *A. wollastoni*.

Whether this enhanced local advective flux increases metabolic activity (net oxygen flux) depends on the extent to which diffusion limitation occurs at the tissue surface, that is, whether oxygen consumption exceeds the rate of resupply across the CBL in absence of ciliary flow. To evaluate this, we compared the characteristic timescales of oxygen consumption and transport using the Damköhler number (Da), a dimensionless parameter expressing the balance between metabolic demand and mass-transfer supply (for Da calculation and derivation, see Supplementary Methods, Eqs. 1–4^30,67–69^). The metabolic demand corresponds to the time needed for the coral to consume all the oxygen present in its immediate surroundings, which we determined through volumetric oxygen consumption in vials. The mass transfer is expressed by the diffusive timescale, which is the time required for oxygen to cross the CBL (Supplementary Methods, Eq. 2). Here we assumed diffusion through the upper CBL and stirring in the ciliary vortices (lower CBL). When Da approaches unity, diffusion through the boundary layer is sufficient to sustain metabolism. In contrast, when Da « 1, diffusion limitation occurs. Interestingly, for *A. wollastoni*, we calculated Da = 0.77 suggesting that diffusion of oxygen is sufficient or very close to meet metabolic demands. The high surface area in combination with flow through the colony allows for quick diffusive transport through the thin boundary layer. *A. wollastoni* thus does not rely strongly on active transport via ciliary flow to increase oxygen supply and cilia are then primarily used to redistribute solutes locally. In sharp contrast, we calculated Da = 0.05 for *Stichopathes* sp., indicating that in this species diffusion-limitation occurs (Supplementary Methods, Eq. 4). This is consistent with measured oxygen profiles where the absence of ciliary flows results in non-detectable oxygen concentrations at the tissue surface. We note that the estimation of the Da number relies on the respiration rates determined in closed vials. Although mixing in the vials was promoted by regular inversions, diffusion-limitation and boundary layer formation cannot be fully excluded, potentially leading to an underestimation of respiration rates and consequently slightly overestimated Da numbers.

Together, these results indicate that the functional role of ciliary flow differs between species. It enhances net fluxes in *Stichopathes* sp., but in *A. wollastoni* it primarily redistributes solutes locally, without increasing overall supply under well-ventilated conditions. This raises the question why *A. wollastoni* would invest energy in generating ciliary flows that do not enhance oxygen supply.

### Trade-off between colony morphology and ciliary function in coral-seawater exchange

To answer this question and elucidate potential alternative roles of ciliary motion, we estimated the clearance rate in both species, defined as the volume of seawater passing over the coral surface, per unit time. Clearance rates were ~3.7 times higher in *A. wollastoni* than in *Stichopathes* sp. (Fig. S1), consistent with its ~2.8 times higher surface-to-volume ratio (12.7 ± 0.7 vs 4.5 ± 0.2 mm^−1^). This ratio increased to 3.3 when polyps were excluded, indicating that polyps allow for compensation, at least in part, for the reduced surface of exchange of *Stichopathes* sp.

**Fig. 6:**
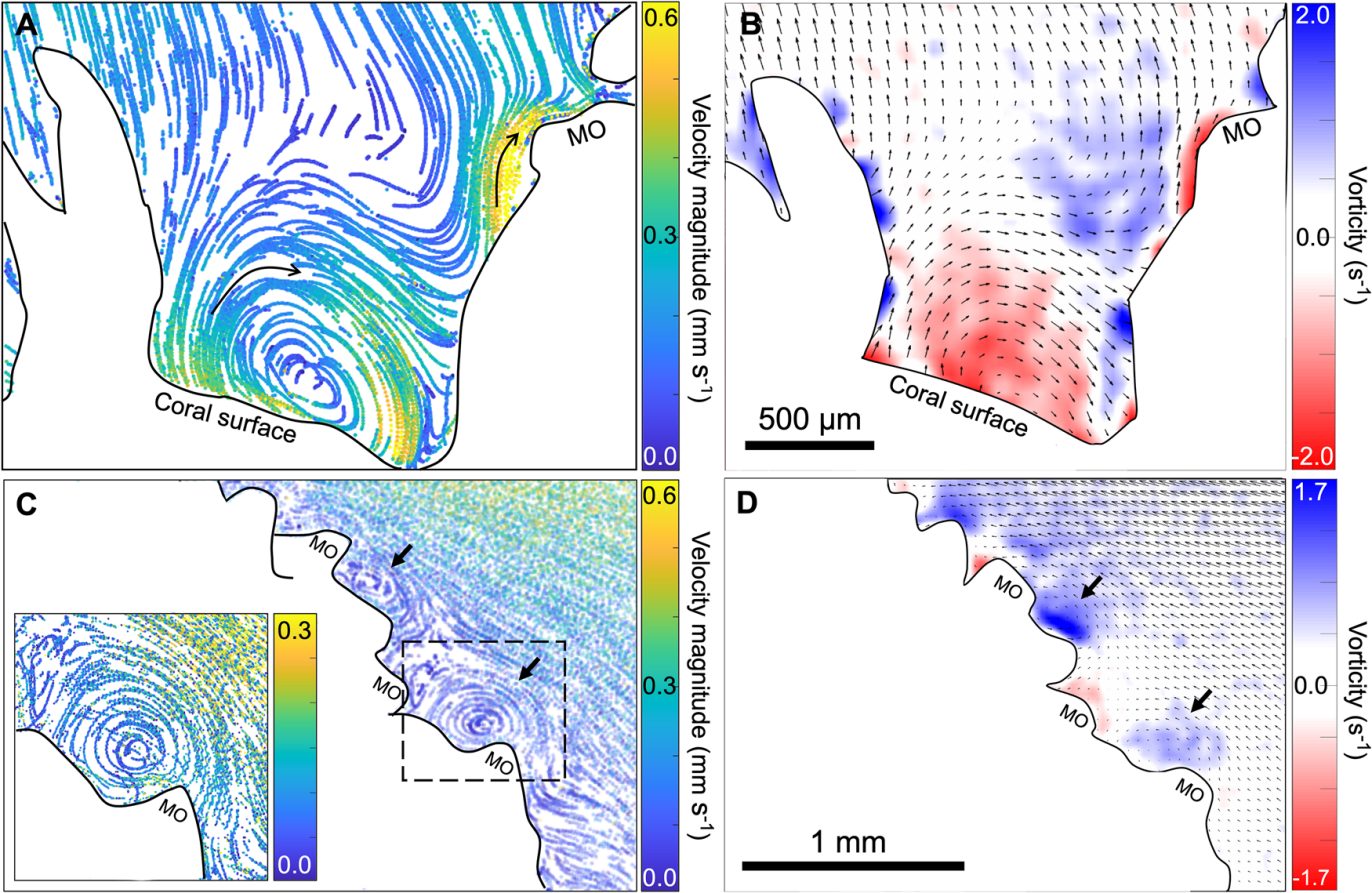
Vortical flow field characterization, in Stichopathes sp. **A**, **B** and A. wollastoni (**C**, **D**).** A**,** C** Velocity magnitude varies within the vortices and accelerates close to the ciliated tissue surface (Movie S4). **B**, **D** High vorticity regions cluster near the tissue surface. Inset in **C**: close-up view on the vortex in *A. wollastoni*. Curved black arrow: flow direction; Straight black arrows: vortex location, MO Mouth Opening.

These clearance rate estimates capture transport immediately above the tissue and mouth openings, but solutes and particles must also traverse the surrounding CBL where ciliary-induced vortices enhance mass transport. μPIV revealed vorticity of the ciliary vortices of ~2 s⁻¹ (one rotation ~6 s), generating tangential flows that deliver particles across the boundary layer in ~1 s, an acceleration of more than two orders of magnitude compared to diffusion (~2 min) (Fig. 6, Movie S4; for transport timescale calculations, see Supplementary Methods, Eq. 2). In *A. wollastoni*, vortices were uniformly distributed, rapidly transporting particles to tissue and mouth openings (Fig. 3). We also observed particles traversing the dense cilia layer (Movie S5), a mechanism that warrants further investigation. In *Stichopathes* sp., ciliary activity at mouth openings was also very active and ingestion of microalgae was observed. On top of that, the large mouths and long tentacles of its polyps allow this species to efficiently capture larger prey like *Artemia* (Movie S6), suggesting that cilia likely play a role in the capture of small food items, while tentacles (and nematocysts) are used for larger food items like *Artemia*. Both feeding strategies are likely used in all species, but food item sizes will ultimately depend on mouth size. The branched *A. wollastoni* achieves high particle clearance through its extensive exchange surface (beneficial for feeding), reducing reliance on mass transport enhancement via cilia and allowing it to have small polyps and mouth because the ingestion of small food items suffices. On the other hand, the whip-like *Stichopathes* sp. has a smaller surface of exchange and has larger polyps and mouths that allow it to optimize food acquisition by capturing smaller (via ciliary flow) and larger (via tentacle activity and nematocysts release) food items. These observed trade-offs between coral colony morphology, polyp size, and mass transport enhancement by cilia suggest that cilia-driven flow may serve as a compensatory mechanism for limited exchange surface. Investigating ciliary flow within coral gastric cavities, where geometry and ciliary activity shape internal transport, could reveal the colony’s full mass transfer, both at the seawater interface and within the coral.

### Ciliary activity and skeletal structures regulate mass transport within the gastrovascular system

**Fig. 7:**
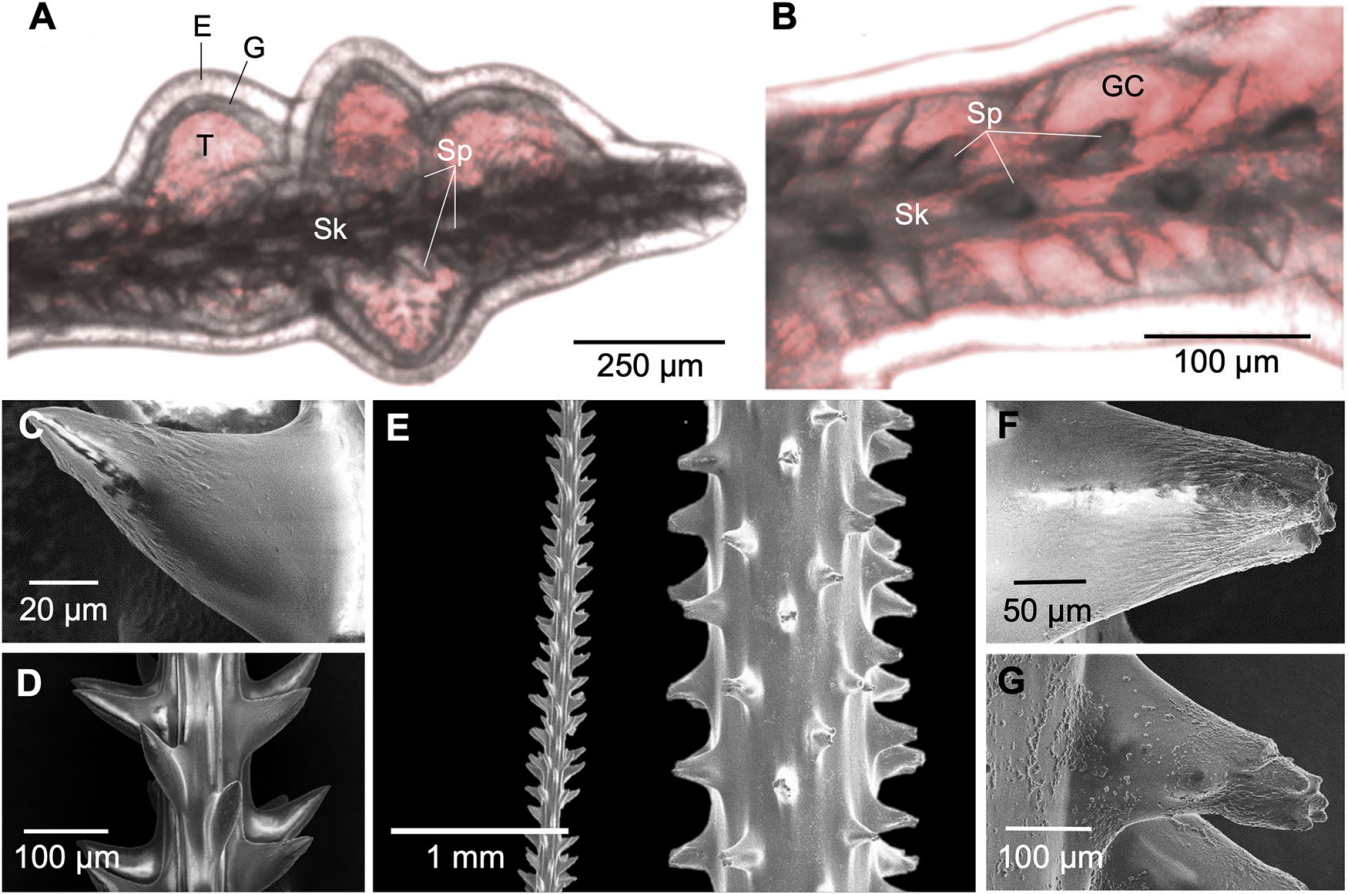
Internal fluid dynamics are shaped by ciliary flow and the arrangement of the spines creating internal cavities across polyps. **A**, **B** Ciliary flow creates particle movement inside the tentacles of the polyp (**A**) and in the coenosarc (biomass connecting two adjacent polyps) (**B**). Red color indicates area in which particles were observed to move. Particle movement was bidirectional (Movies S7–S9). The spiny chitino-proteinaceous skeleton is visible. E: ectoderm, G: gastroderm, T: tentacle, Sk: skeleton, Sp: spines, GC: gastrovascular canal. **C**–**G** SEM secondary electron micrograph images of the skeleton and spines of *Antipathella wollastoni* (Left panel) and *Stichopathes* sp. (Right panel).

We used high-speed videography to directly visualize flow patterns inside the gastrovascular system of antipatharian corals. These recordings revealed that ciliary activity is modulating hydrodynamic circulation within the gastrovascular cavities and tentacles of *A. wollastoni* (Fig. 7A, B; Movies S7 and S8) and in the tentacles of *Stichopathes* sp. (Movie S9). Moving tracer particles (non-motile *Nannochloropsis* sp.) revealed bidirectional flow within gastrovascular cavities (Movie S7). Larger particles (>500 µm diameter), most likely detrital particles, were also observed inside tentacles (Figure S6, Movie S10). Particle tracking (PTV) showed internal flow velocities of 0–300 µm s^−1^, which is slightly lower than external cilia-driven flows, but comparable to those measured in *Acropora* specimens (160–320 µm s^−1^)^53^. In *Stichopathes* sp., bidirectional flow was observed in the tentacles but could not be seen in the gastrovascular cavities due to optical constraints from thicker tissues. To the best of our knowledge, these results represent a first direct observation of internal flow in hexacorals using naturally occurring particles in combination with natural ingestion (probably induced by ciliary flows), extending previous indirect inferences based on particles or dye injections through the oral opening of scleractinian corals^52,53,70^. These observations suggest that ciliary flow allows connection between polyps in antipatharian corals and facilitates digestion across the entire colony. The gastrovascular system appears to function as an effective circulatory system that facilitates rapid intracolonial transport.

We attribute slower internal flow velocities, as compared to external flow velocities, to internal viscous dissipation, geometric confinement, and boundary interactions with internal architecture. The spatial organization of internal flows is closely linked to the skeletal scaffold composed of a main stem bearing spines (Fig. 7C–G). The geometry of the internal space is defined by spines measuring ~105 and 257 µm attached to stems with an inner radius of 120 and 897 µm for *A. wollastoni* and *Stichopathes* sp., respectively. This effectively partitions the gastrovascular cavity into distinct flow domains. In the absence of spines, the outer tissue layer would sit directly on the skeleton, eliminating internal cavities that facilitate circulation. Thus, the skeletal scaffolding shapes the internal hydrodynamic environment and influences transport efficiencies. A coupling between structural morphology and internal fluid flow is a recurring feature across benthic metazoans with internal canal systems, including sponges, bryozoans, and certain cnidarians (e.g., refs. ^71–73^). In *A. wollastoni* and *Stichopathes sp*., the dimensions, spatial arrangement, and orientation of spines determine cavity volume and modulate both transport directionality and exchange efficiency. This underscores the essential role of antipatharian spines in structuring the internal environment allowing for intracolonial connectivity.

## Conclusion

Taken together, our findings show that ciliary activity governs mass transport in antipatharians not only at the colony-seawater interface, but also within the gastrovascular system. Whereas colony-scale morphology modulates the efficiency of external exchanges, the internal skeletal architecture defines the flow dynamics of gastrovascular canals. This points towards a common theme: ciliary beating interacts with structural features to regulate transport across scales. Ciliary flow fulfills a dual function in antipatharians, by (1) mediating exchanges (ventilation and feeding) with the surrounding seawater and (2) ensuring intracolonial connectivity through internal circulation. Our results further suggest that antipatharians optimize ciliary flow in a morphology-dependent manner. The unbranched *Stichopathes* sp. relies strongly on ciliary flow to ventilate at the colony-seawater interface and to support feeding, compensating for its reduced exchange surface. In contrast, the branched *A. wollastoni* depends less on ciliary activity for ventilation, benefitting from a larger exchange surface, yet still employs ciliary motion for local redistribution of solutes and compounds. Nevertheless, its dense branching restricts exchange or ventilation within the colony interior, which may become limiting under specific conditions like low-flow or low-oxygen, scenarios increasingly common at mesophotic depths under global change.

Our experiments were conducted under laminar, low-flow conditions. Under turbulent background flow, however, ciliary vortices are likely to interact with ambient turbulent regimes, leading to more complex and potentially intermittent transport dynamics. Such interactions may enhance or disrupt local mixing, alter CBL thickness dynamically, and thereby modulate mass transfer rates at the tissue-fluid interface. Investigating these coupled processes under realistic turbulent conditions represents an important avenue for future work. Cilia thus emerge as central to black coral respiration, feeding, and intracolonial transport, linking microscale hydrodynamics to the evolution of black coral form and function. Ultimately, this inherent link between ciliary function and colony morphology may serve as a predictor of coral resilience, but establishing this will require broader characterization of these traits, across black corals but also reef-building scleractinians.

## Methods

### Coral collection

The arborescent *Antipathella wollastoni*^74^ is widespread across Macaronesia and occurs from 25 m to more than 1000 m, forming dense mesophotic assemblages^75–77^. The whip-shaped *Stichopathes* sp. occurs in the Canary Island archipelago at depths from 30 to 150 m and is known to be densely abundant around the island of Lanzarote^76^. At 70 m depth near Gran Canaria, its distribution was sparse and patchy, likely due to the sandy substrate (Fig. S7).

*A. wollastoni* fragments were collected by scuba diving at 35 m depth on April 1st, 2025 (28°01'56.0“N 15°22'32.0“W), and *Stichopathes* sp. fragments by technical diving (rebreathers) at 73 m depth on April 7^th^, 2025 (28°02'23.3“N 15°21'59.2“W). In total, fourteen ~10 cm-long fragments were collected per species, on the East coast of Gran Canaria. Fragments were cut from the colony, placed in ziplock bags with seawater of the collection site, and transported in cool boxes to the Parque Científico Tecnologico Marino de Taliarte (Telde, Gran Canaria), within 1 h. In the laboratory, fragments were cut to obtain two ~5 cm-high fragments per colony, individually tagged, attached using underwater epoxy resin (Holdfast, Aquarium Systems, France) and placed in an open-circuit aquarium (80 L, salinity 36.8‰) at 19 °C, corresponding to the temperature at both collection sites. Maintenance in aquaria was conducted according to the protocol successfully used in earlier studies^78,79^.

### Light microscopy and cilia beating frequency

A light microscope (Leica DM 1000) was used to capture the cilia and particle movements inside gastrovascular cavities. A custom-built chamber was constructed on a standard microscope glass slide with scotch tape. The dimensions were optimized to accommodate a ~ 1 cm fragment for both species. It allowed full submersion of the sample in seawater while minimizing the distance between the tissue surface and glass, thereby facilitating optimal focus on ciliary structures.

High-speed videography (420–720 frames per second) was conducted using a light microscope mounted with a Teledyne FLIR IIS Blackfly camera, enabling resolution of individual cilium stroke dynamics. To quantify beating frequency, background subtraction was performed in ImageJ^80^, by generating an average intensity projection and subtracting it from the original image sequence. A subset of the sequence was then extracted by selecting a small rectangular region of interest (ROI; approximately 100–200 × 20 pixels) where ciliary motion was clearly visible and well-defined. Temporal intensity profiles for each pixel within the ROI were computed using a custom MATLAB script. The intensity profiles reveal characteristic wave-like patterns associated with ciliary activity (Fig. S2A). Then, power spectral analysis of these time series was conducted, with the dominant frequency peak corresponding to the average ciliary beating frequency (Fig. S2B).

Additional microscopic observations, with colors, were performed using an Olympus BX53 research-grade microscope equipped with ultraviolet (UV) and infrared (IR) fluorescence excitation filter sets, in combination with differential interference contrast (DIC/Nomarski) optics. The instrument allowed magnifications ranging from 10X to 63X, with the 63X objective operated under oil immersion.

### Coral morphometrics, clearance rate

Species morphologies were compared by taking photographs with a scale, using an Olympus Tough TG-5 (Japan). Photos were taken to be able to characterize the overall morphology of fragments and polyps. Image analyses were performed using ImageJ^80^. Surface and volume of polyps and fragments were estimated by considering tentacles as cones and mouth and stem as cylinders. Differences were made between the four lateral and the two sagittal tentacles, the latter typically being longer than the others. To account for intraspecific variability in morphometrics, seven fragments were considered per species, and their measurements were averaged to characterize the whole species. All morphological measurements were integrated to estimate total surface area, per polyp surface-to-volume ratio and total surface-to-volume ratio, for both species.

To account for differences in overall morphologies, accounting for the ramifications in *A. wollastoni*, clearance rate ($Q$) was estimated. It is defined as the volume of seawater passing over the coral surface, per unit time, and is calculated as $Q=v\times {SA}$, where $v$ is the velocity of seawater at the tissue surface and ${SA}$ is the surface area of a 5 cm-high fragment. Measurements were taken on seven coral fragments per species.

### Flow chamber experimental setup

A 3D-printed flow chamber (22.5 × 4.0 × 4.2 cm) was designed to accommodate the fragments during all experimentations (Fig. S8). A standard glass microscope slide (7.6 × 4.0 cm) was mounted on the front face of the chamber to enable optical access to the specimen with minimal reflections. The top of the chamber was left open to allow vertical access for microsensor positioning. Converging and diverging conical sections were incorporated at the outlet and inlet, respectively, and connected to a water pump (Ismatec, Switzerland) via 0.9 cm-diameter silicone tubing. To ensure laminar and homogeneous flow within the chamber, a < 1 mm mesh filter and a 3D-printed diffuser with 0.2 cm-diameter holes were installed at the inlet.

### Flow-field imaging: particle (image/tracking) velocimetry

The coral fragment was placed inside the flow chamber, filled with seawater from the coral’s culturing tank. Prior to measurements, the specimen was allowed to acclimate to the flow conditions for a minimum of 20 min. Flow fields around the coral were resolved using Particle Image Velocimetry (PIV) and Particle Tracking Velocimetry (PTV). While tracer particles such as green fluorescent polymer microspheres are commonly used to seed the flow, pilot experiments showed that *Nannochloropsis* sp. microalgae, used to enrich *Artemia* and rotifer cultures for coral feeding, provided an ideal alternative. These microalgae exhibit key properties for PIV: appropriate size range (2–3 µm diameter), neutral buoyancy, lack of motility, and natural autofluorescence that is excited with blue light. As a result, all PIV experiments were conducted using *Nannochloropsis* sp. cultures. Notably, even at high concentrations, microalgae seeding did not induce mucus production by the coral.

Illumination was provided by a 5 W pulsed laser diode (LD-PS/5, Optolution GmbH, Germany), emitting a thin light sheet at 450 nm. Laser height was manually adjusted to ensure that the entire field of view is covered. Flow visualization within the light sheet was achieved using two FLIR Grasshopper3 cameras (USA) equipped with different magnification optics: (i) a fixed focal length lens (Fujinon f/1.4 - f/22, 12.5 mm, Fujifilm, Japan) for low-magnification imaging (~10 × 10 cm²), and (ii) a long-distance microscope (K2 DistaMax with CF2 objective, Infinity Photo-Optical Co., USA) at f/5.6 for high magnification (~5 × 5 mm²). Image sequences were acquired at 38 or 76 fps, based on flow speed or camera magnification. To isolate luminescence emission, a yellow Lee filter foil (LEE filters, UK) was placed on the inner glass wall of the flow chamber. To prevent reflections and reduce background noise, matte black aluminum foil was applied to all reflective surfaces. Camera settings, laser and recordings were controlled using FlyCapture2 software (v2.13.3.61).

To simultaneously resolve flow fields and oxygen concentration around the coral fragment (sensPIV), emulsion-based oxygen-sensitive PtTFPP nanoparticles were used following procedures from refs. ^49,50^. For the experiments, 1.5–3 mL of the oxygen-sensitive nanoparticle dispersion was added, giving a nanoparticle concentration of 4.2–8.4 µg mL⁻¹ based on the magnification used.

Illumination of the nanoparticles was achieved using the same pulsed laser diode used for PIV imaging (LD-PS/5, Optolution GmbH, Germany), employing the frame-straddling method to quantify lifetime-proportional luminescence^66^. Images were recorded using a FLIR Grasshopper3 camera (USA) equipped with interchangeable lenses with different magnification.

Prior to experimental measurements, the oxygen-sensitive nanoparticles were calibrated using two reference solutions with known oxygen concentrations. For each experiment, 100–300 image pairs were captured with the following settings: 38 or 75 fps, 0.05 ms pulse width and camera gain set to 10–20. Post-processing of image stacks was performed using a custom MATLAB script to extract oxygen concentration signals (“sens”), while flow fields were resolved using ImageJ^80^ and the PIVlab toolbox^81^. Additionally, Bulk oxygen levels were monitored with an electrochemical oxygen sensor^82^.

### Oxygen profiles with active and arrested ciliary flow

The microsensor tip was positioned at the coral surface or at the bottom of the mouth, using a motorized micromanipulator. Dissolved oxygen concentration profiles were obtained by incrementally retracting (moving upward) the microsensor from the initial contact point in 100 and 20 µm steps (for *Stichopathes* sp. and *A. wollastoni*, respectively), until background oxygen levels were reached. Profiles were acquired for both species at two anatomical locations: (1) at the tissue surface adjacent to the mouth opening, and (2) inside the mouth opening. When feasible, profiles were repeated at the same location and averaged to improve accuracy. In some instances, tentacle movement or closure of the mouth prevented repeated measurements.

To assess the influence of ciliary motion on oxygen distribution, profiles were compared under conditions with active and arrested cilia. Ciliary motion was inhibited by applying sodium orthovanadate 0.1 M (Na₃VO₄; Sigma-Aldrich), a reversible ATPase inhibitor previously used to suppress ciliary beating in scleractinian corals^30,49^.

### Bulk oxygen consumption

Bulk oxygen consumption rates were measured for both species in the presence and absence of sodium orthovanadate, to control for its potential effect on overall metabolism. In total, four fragments of *Stichopathes* sp. and two of *A. wollastoni* were tested in both treatments (with and without sodium orthovanadate). Genotypes tested were identical across treatments, and identical genotypes were tested simultaneously, to remove potential effects of metabolic activity differences across treatments. Fragments of *Stichopathes* sp. were on average 7.25 mm long (3.9 polyps) and fragments of *A. wollastoni* on average 13.94 mm long (14.3 polyps). Fragments were incubated in 12 mL glass exetainers filled with filtered seawater from the collection site. One vial per test was filled with only filtered seawater, to account for background microbial respiration (control). To maintain stable environmental conditions (e.g., temperature, light), the exetainers were submerged in the same aquarium system used for coral culturing. The vials were gently inverted at regular intervals to ensure homogenous distribution of dissolved oxygen and temperature. Oxygen concentration was measured every 60 min for a total time between 317 and 455 min, using a FireSting GO2 oxygen meter (PyroScience GmbH, Germany). Each time series included at least 5 time points. Oxygen concentrations were recorded in % air saturation and then converted into µmol L^−1^ min^−1^ using known solubility at salinity 37 and temperature 19 °C. Oxygen consumption rate (µmol min^-1^) was extracted from the slope of oxygen concentration with time, accounting for the volume of seawater in the vial. Oxygen concentration rates were normalized by the surface area of the fragment, estimated from image analysis. Final units are µmol O_2_ min^−1^ cm^−2^. Other normalization methods were also tested and compared: normalization by length of the fragment (µmol O_2_ min^−1^ cm^−1^), and by polyp number (µmol O_2_ min^−1^ polyp^−1^).

### Statistics and reproducibility

All statistical analysis were performed with the software R version 4.4.0^73^.

The effect of ciliary flow (active/inactive) on diffusive and advective fluxes were tested using t-tests (Table S2). The effect of sodium orthovanadate addition on the coral metabolism was tested by performing two-ways ANOVAs, with main effects (species and treatments) and interaction effect (i.e., if the sodium orthovanadate has different effects on the two species). No significant effect of sodium orthovanadate on respiration rates was observed in either species (Fig. S9, Table S3), supporting the use of sodium orthovanadate to arrest ciliary flow in antipatharians, without confounding metabolic measurements.

### Reporting summary

Further information on research design is available in the Nature Portfolio Reporting Summary linked to this article.

## Supplementary information

Transparent Peer Review file

Supplementary Information

Description of Additional Supplementary files

Supplementary Data

Movie S1

Movie S2

Movie S3

Movie S4

Movie S5

Movie S6

Movie S7

Movie S8

Movie S9

Movie S10

Reporting Summary

## Data Availability

The authors declare that all data supporting the findings of this study are available within the paper, in the Supplementary Information and in the Supplementary Data.

## Code availability

The code for the basic processing of sensPIV data is publicly available on Github: https://github.com/SoerenAhmerkamp/sensPIV.git
