# Supplementary material for: Ciliary flow and morphology shape mass transport at the surface and within gastrovascular cavities of black corals
Source: Commun Biol. 2026 Jun 30;9:876. doi: 10.1038/s42003-026-10531-2 (PMC13319206; doi:10.1038/s42003-026-10531-2)
Supplement: Supplementary file 2 — Supplementary Information [file 42003_2026_10531_MOESM2_ESM.pdf]

Supplementary Materials for

Ciliary flow and morphology shape mass transport at the surface and within gastrovascular cavities of black corals

Mathilde Godefroid *et al.*

*Corresponding authors: Mathilde Godefroid, godefroid.mathilde1@gmail.com;
Soeren Ahmerkamp, soeren.ahmerkamp@io-warnemuende.de

This PDF file includes:

1. Supplementary Figures and Tables:

   Figs. S1 to S9

   Tables S1 to S3

2. Supplementary Methods

1. Supplementary Figures and Tables

A		*Stichopathes sp.*		*A. wollastoni*	
		Mean	se	Mean	se
Polyp	SA (mm^2)	39.69	3.5	2.92	0.24
	Volume (mm^3)	6.61	0.64	0.17	0.02
	Density (polyp cm^{-1})	5.23	0.21	8.92	0.37
	Density (polyp cm^{-2})	11.25	1.21	64.8	5.07
	SA/V	6.25	0.41	17.63	0.72
Branch/Stem	Diameter (mm)	1.51	0.26	0.45	0.09
	Branching density (branch cm^{-1})			8.59	0.71
	Inter-branch spacing (mm)			2.82	0.29
Fragment	SA/V, with polyps	4.47	0.18	12.66	0.66
	SA/V, without polyps	2.78	0.23	9.28	0.59
	Clearance rate (L h^{-1} 5 cm-high $frag^{-1}$)	1.05		3.84	

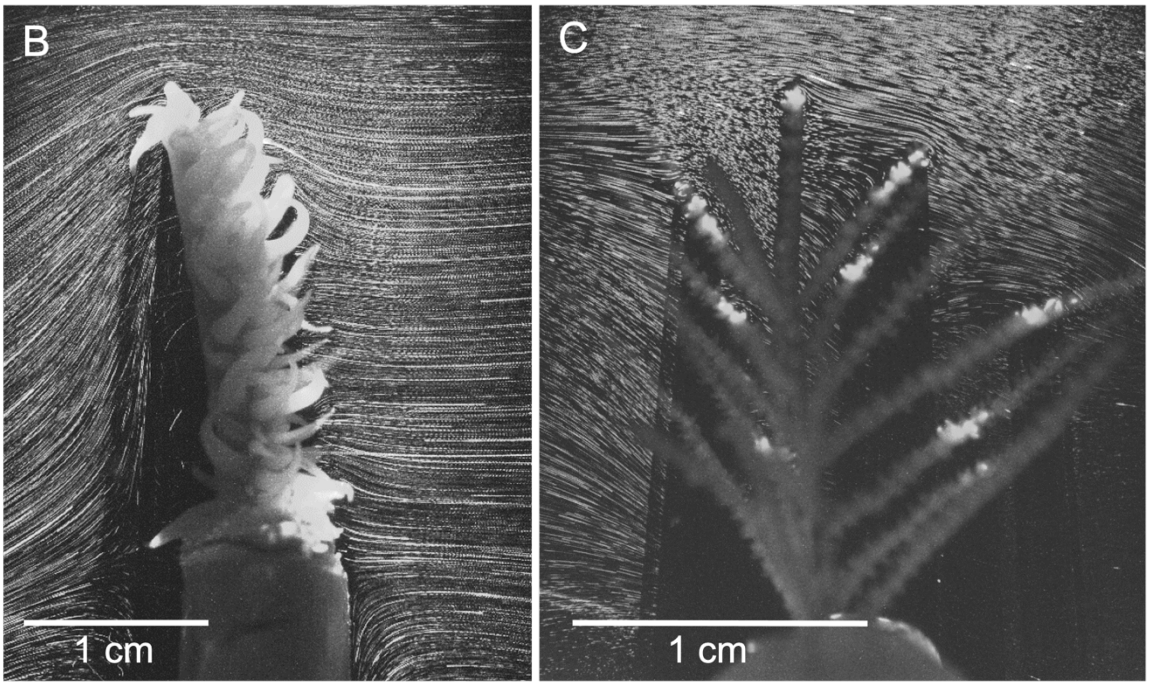

Fig. S1. Theoretical surface-to-volume (SA/V) ratios and clearance rate of *Stichopathes* sp. and *Antipathella wollastoni*, as estimated from morphological measurements. A Comparative table of the main morphological characteristics of the two species and clearance rate estimation. All morphological measurements are summarized in Table S1 and full morphometrics dataset is provided as Supplementary Data. **B, C** Maximum intensity projections of *Stichopathes* sp. and *A. wollastoni* fragments exposed to flow.

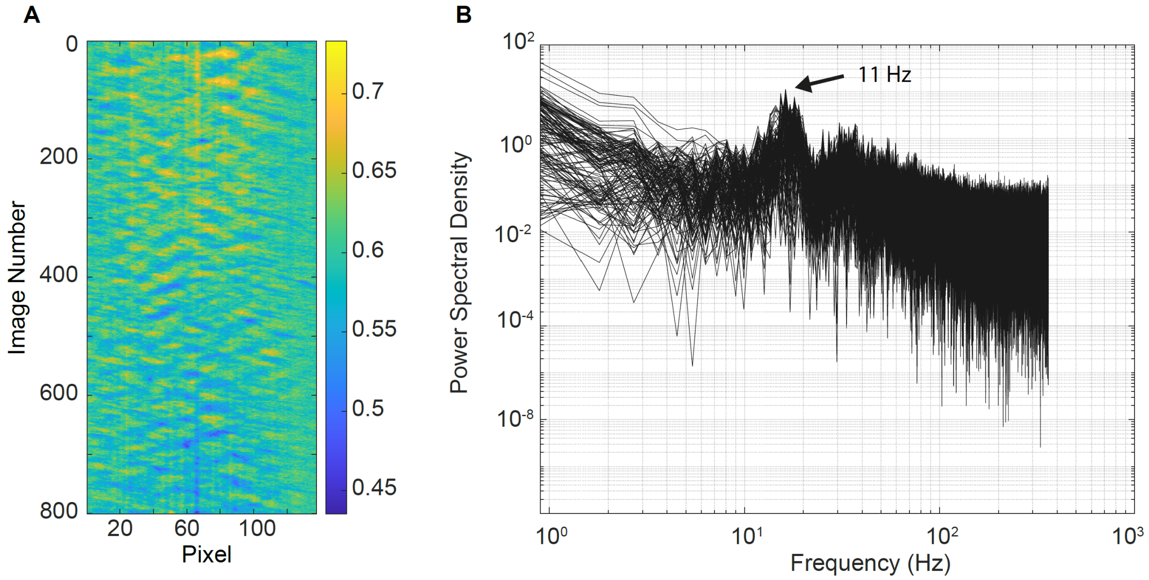

Fig. S2. A Changes in intensity levels induced by the cilia beating. On the x-axis, the pixels of the cut ROI are depicted. **B** Power spectrum density of each pixel. The peak represents the average stroke frequency of the cilia.

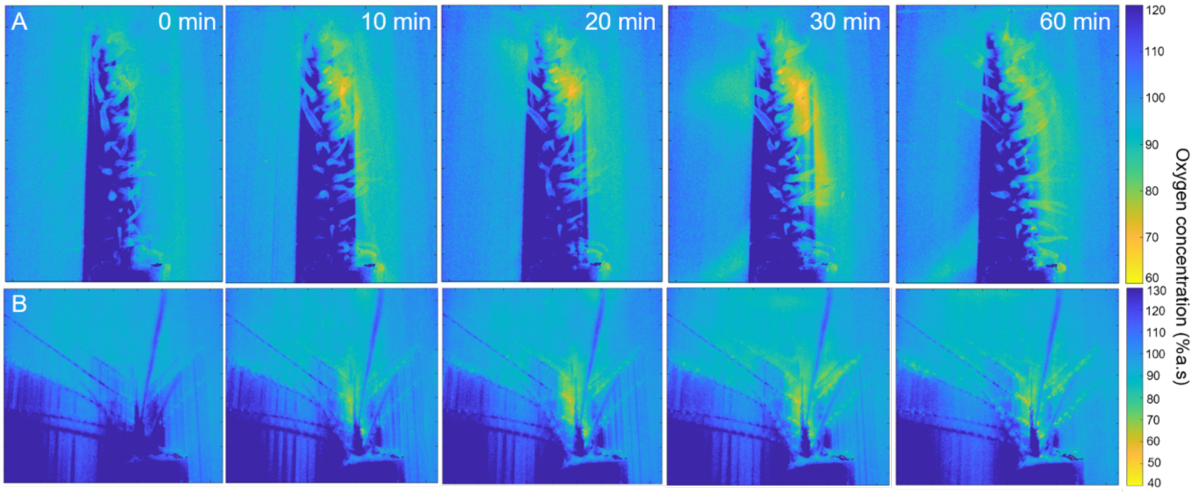

Fig. S3. Oxygen concentration (SensPIV) through time in the dark and in the absence of flow for **A** *Stichopathes* sp. and **B** *Antipathella wollastoni*. The coral fragment was not masked. The dark blue stripes show the areas of the fragment that are shaded by the laser sheet.

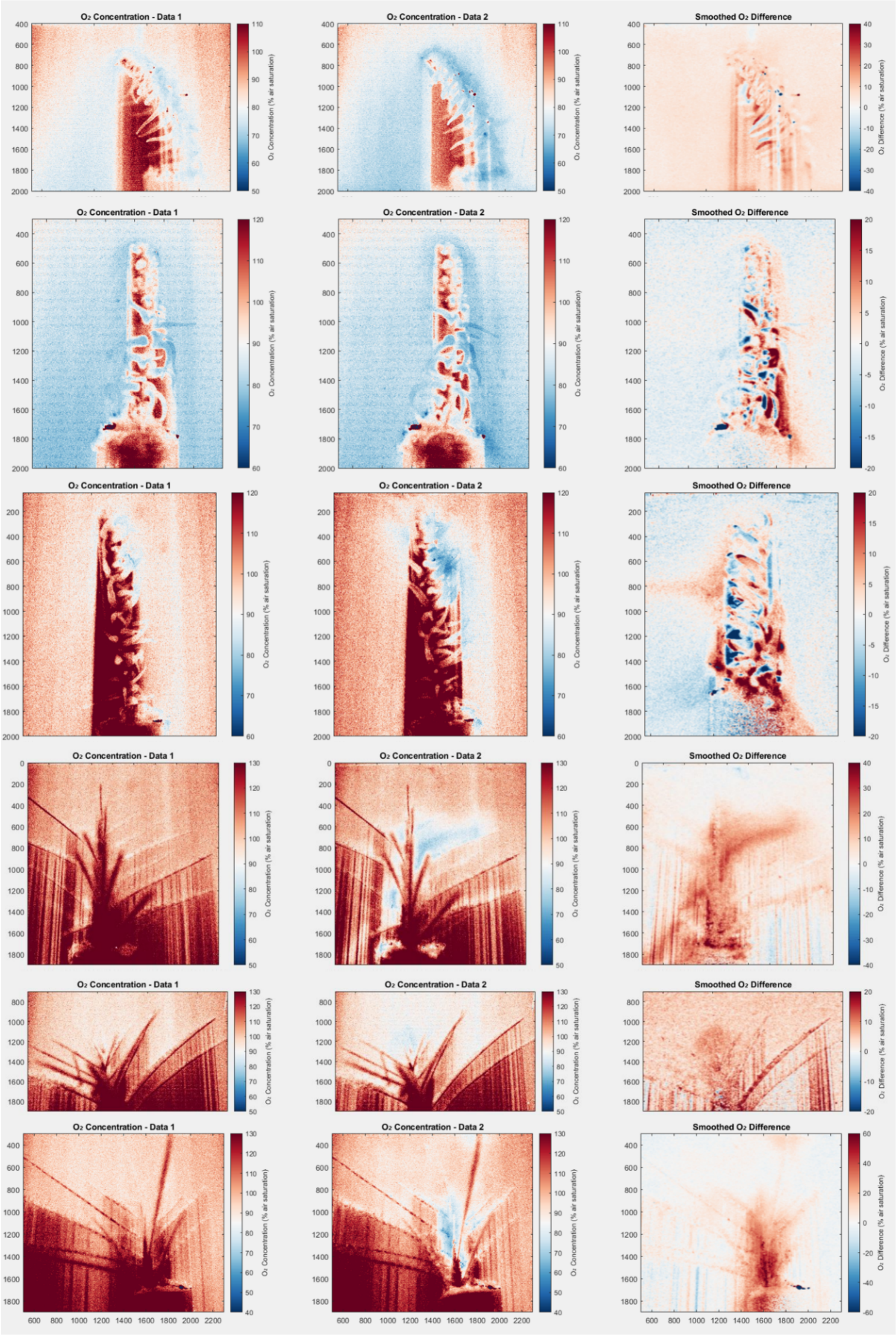

Fig. S4. Overview of the sensPIV results of three fragments per species, showing the variability in oxygen consumption between fragments.

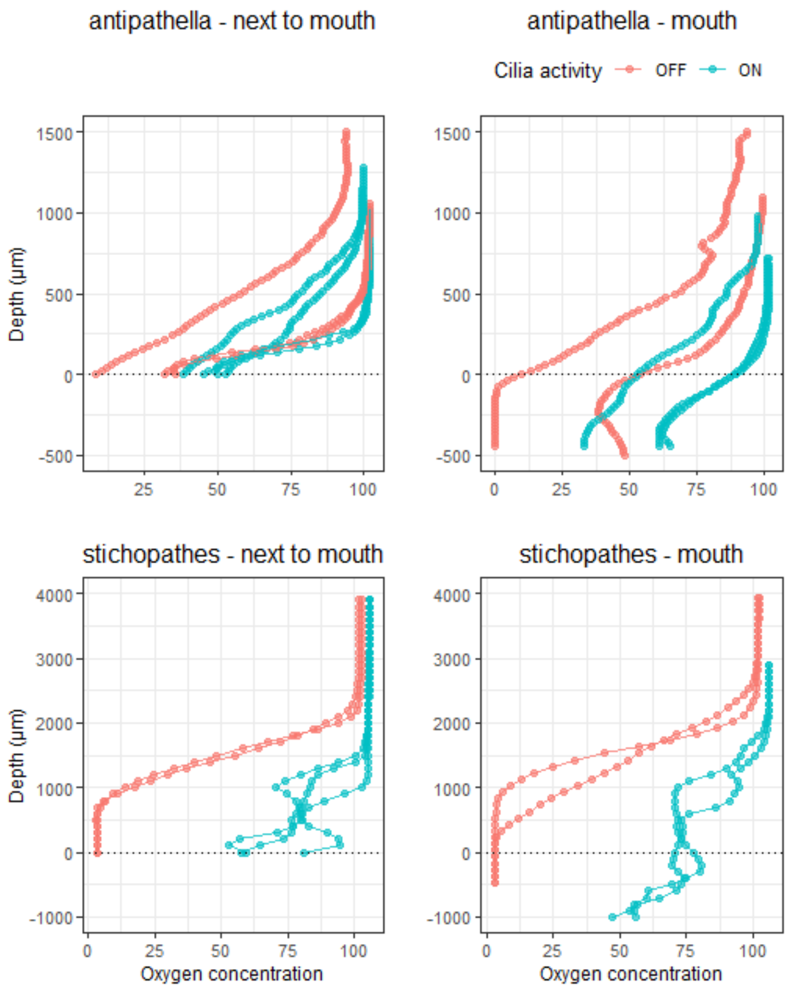

Fig. S5. Overview of oxygen concentration profiles across location and species, with active (green) and inactive (red) cilia. Source data underlying graph can be obtained from Supplementary Data.

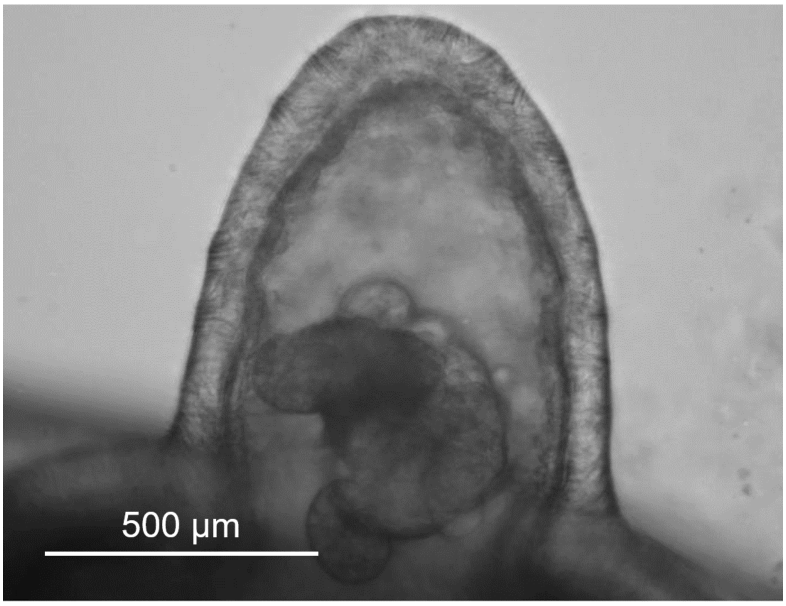

Fig. S6. Tentacle of *A. wollastoni* containing a large internal colloid (light microscopy).

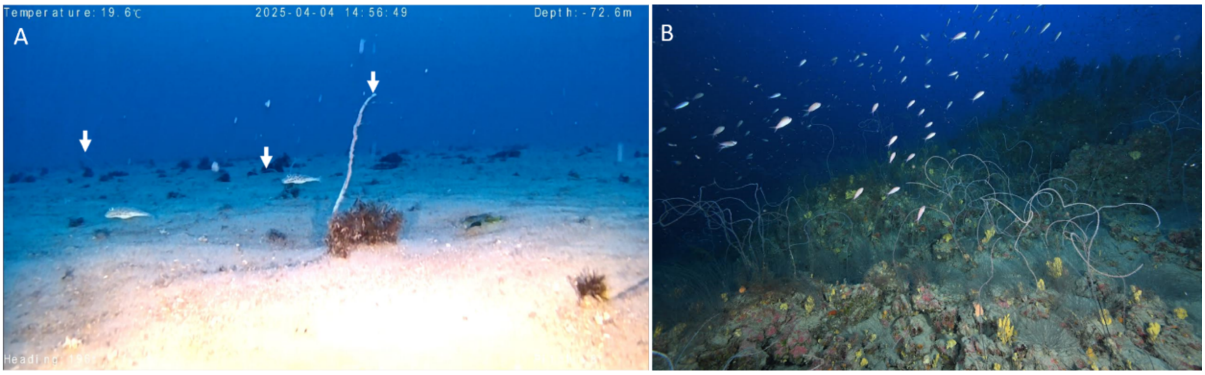

Fig. S7. A Photograph of the site of collection of *Stichopathes* sp. at 73 m depth (28°02'23.3"N 15°21'59.2"W). White arrows depict individual colonies on the seafloor. **B** Photograph of the black coral forest around Lanzarote, depicting high density of colonies of *Stichopathes* sp. in the forefront and of *Antipathella wollastoni* in the back. Photo credit: Fernando Espino Rodríguez.

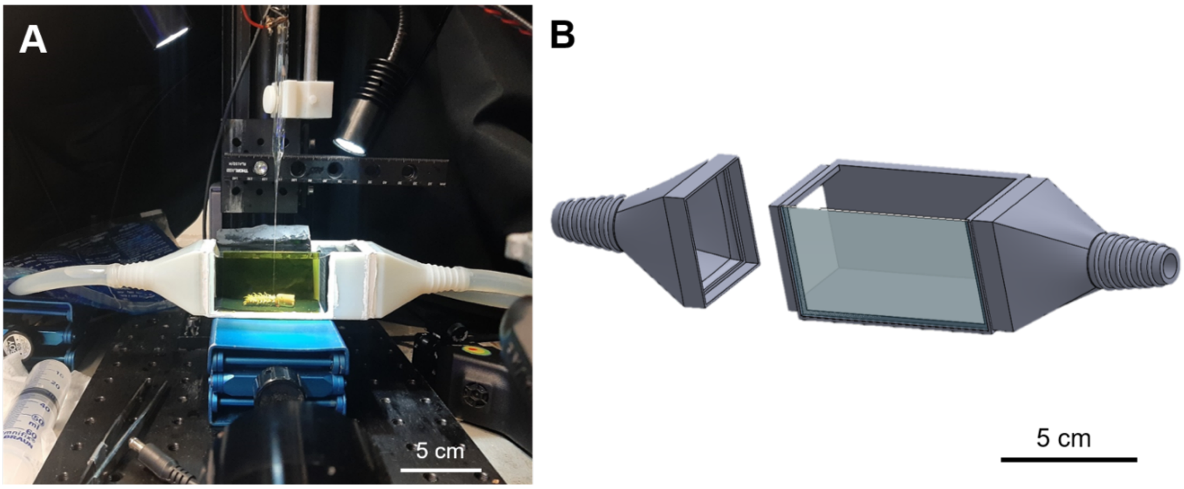

Fig. S8. A Photograph of the custom-made flow chamber used for all experimentations. The flow chamber is connected to a pump via silicon hoses and a homogenizer is placed at the inlet, to get homogeneous flow inside the chamber. A coral fragment lies down on the bottom and a microsensor is visible. **B** Sketch of the 3D printed flow chamber.

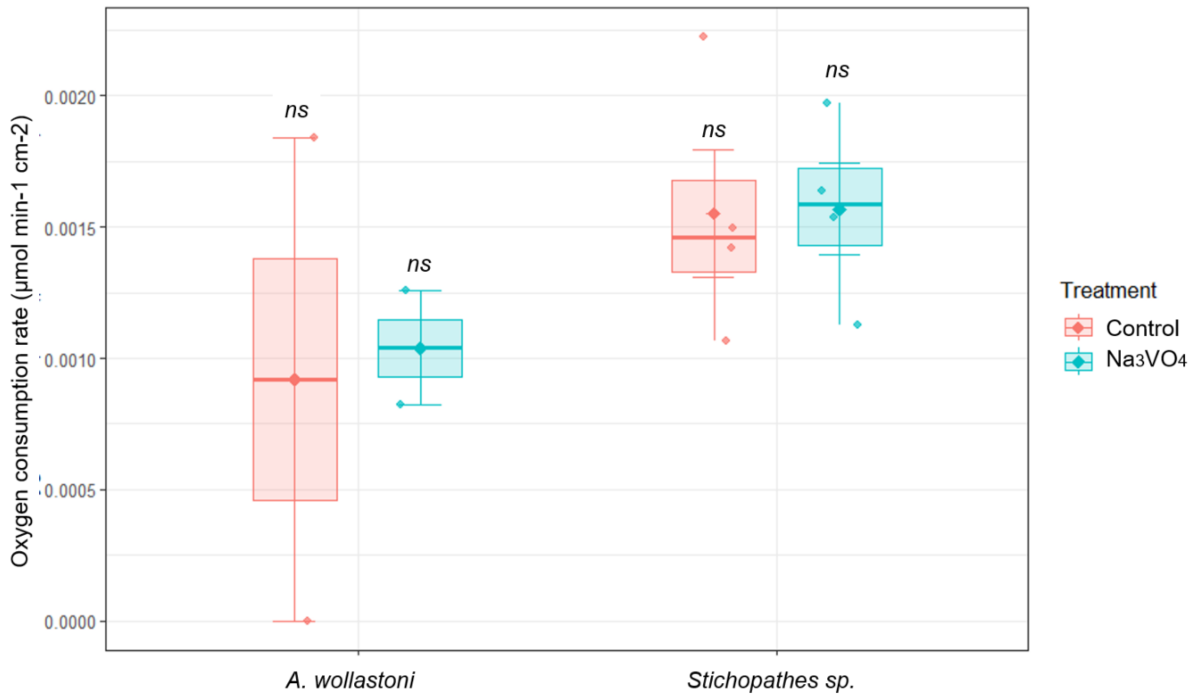

Fig. S9. Oxygen consumption rate per unit surface (µmol min^{-1} cm^{-2}), for *A. wollastoni* and *Stichopathes* sp., with and without addition of sodium orthovanadate (Na_3VO_4) (values provided in Supplementary Data). Two-way ANOVA revealed that main effects (species and treatments) and the interaction (species*treatment) were not significant (*ns*), suggesting no effect of sodium orthovanadate on overall antipatharian metabolism. Normalization per unit length and per polyp number showed differences between species, but the interaction remained non-significant (Table S3). Boxes represent the interquartile range (IQR) with the median (horizontal line). Error bars represent the smallest and largest values within 1.5 x IQR. Source data underlying graph can be obtained from Supplementary Data.

		Stichopathes sp.		*A. wollastoni*	
		Mean	SE	Mean	SE
Sagittal tentacles	Diameter (mm)	1.11	0.08	0.37	0.03
	Radius (mm)	0.56	0.04	0.18	0.02
	Curved length (mm)	4.81	0.35	0.98	0.16
	Total SA (mm^2)	9.74	1.20	0.69	0.15
	Curved SA (mm^2)	8.74	1.05	0.58	0.13
	Volume (mm^3)	1.67	0.31	0.04	0.01
Lateral tentacles	Diameter (mm)	0.81	0.07	0.32	0.03
	Radius (mm)	0.41	0.03	0.16	0.01
	Curved length (mm)	3.33	0.36	0.66	0.08
	Total SA (mm^2)	4.70	0.44	0.40	0.04
	Curved SA (mm^2)	4.17	0.40	0.32	0.04
	Volume (mm^3)	0.57	0.08	0.02	0.00
Mouth	Diameter (mm)	1.18	0.11	0.30	0.02
	Radius (mm)	0.59	0.05	0.15	0.01
	Height (mm)	0.91	0.11	0.33	0.01
	SA (mm^2)	5.53	0.64	0.47	0.04
	Volume (mm^3)	0.98	0.17	0.02	0.00
Polyp	SA (mm^2)	39.69	3.50	2.92	0.24
	Volume (mm^3)	6.61	0.64	0.17	0.02
	Density (polyp cm^{-1})	5.23	0.21	8.92	0.37
	Density (polyp cm^{-2})	11.25	1.21	64.80	5.07
	SA/V	6.25	0.41	17.63	0.72
Fragment	SA/V, with polyps	4.47	0.18	12.66	0.66
	SA/V, without polyps	2.78	0.23	9.28	0.59
	Clearance rate (L h^{-1} 5 cm-high $frag^{-1}$)	1.05		3.84	

Table S1. Summary of the main morphological characteristics of *A. wollastoni* and *Stichopathes* sp., with final calculation of surface-to-volume ratio (all values provided as Supplementary Data). In total, seven fragments per species were used to calculate mean ± se. Curved length: length of the tentacle measured by accounting for its curvature, SA: surface area, Total SA: surface area calculated by including the base of the tentacle (considered to be a disk); Curved SA: surface area calculated without including the base of the tentacle; SA/V: surface-to-volume ratio. Source data underlying table can be obtained from Supplementary Data.

(A) Diffusive flux (µmol cm^{-2} d^{-1})							
Species	Location	group 1	group 2	statistic	df	p	p.adj
A. wollastoni	Mouth	cilia off	cilia on	-0.08	1.16	0.947	ns
A. wollastoni	Next to mouth	cilia off	cilia on	-0.07	3.22	0.948	ns
Stichopathes sp.	Mouth	cilia off	cilia on	4.94	5.90	***0.003***	**
Stichopathes sp.	Next to mouth	cilia off	cilia on	3.06	5.16	0.027	ns
(B) Advective flux (µmol cm^{-2} d^{-1})							
Species	Location	group 1	group 2	statistic	df	p	p.adj
A. wollastoni	Mouth	cilia off	cilia on	-0.98	2.04	0.427	ns
A. wollastoni	Next to mouth	cilia off	cilia on	-1.84	3.68	0.146	ns
Stichopathes sp.	Mouth	cilia off	cilia on	-9.73	4.33	***0.0004***	***
Stichopathes sp.	Next to mouth	cilia off	cilia on	-13.30	5.06	***0.00003***	***
(C) Advective flux 300 µm (µmol cm^{-2} d^{-1})							
Species	Location	group 1	group 2	statistic	df	p	p.adj
A. wollastoni	Coenosarc	cilia off	cilia on	-1.73	4.88	0.145	ns
A. wollastoni	Mouth	cilia off	cilia on	-2.02	2.00	0.181	ns
A. wollastoni	Next to mouth	cilia off	cilia on	-1.76	3.13	0.173	ns
Stichopathes sp.	Mouth	cilia off	cilia on	-3.86	4.14	***0.017***	*
Stichopathes sp.	Next to mouth	cilia off	cilia on	-5.70	5.03	***0.002***	*

Table S2. Summary statistics of t-tests to test for the effect of ciliary flow (on/off) on (A) Diffusive flux in the upper boundary layer; (B) Advective flux, calculated by averaging values over the lower boundary layer; (C) Advective flux, calculated by averaging values 300 µm above tissue surface.

(A) per unit surface (µmol min^{-1} cm^{-2})					
	Df	Sum Sq	Mean Sq	F value	Pr(>F)
Species	1	8.9e-07	8.9e-07	2.497	0.153
Treatment	1	7.4e-09	7.4e-09	0.021	0.889
Species:Treatment	1	7.0e-09	7.0e-09	0.020	0.892
Residuals	8	2.9e-06	3.6e-07		
(B) per polyp (µmol min^{-1} $polyp^{-1}$)					
	Df	Sum Sq	Mean Sq	F value	Pr(>F)
Species	1	9.3-07	9.3e-07	43.5	***0.000171***
Treatment	1	1.0e-10	1.0e-10	0.004	0.952105
Species:Treatment	1	0.0e+0	0.0e+0	0.000	0.988470
Residuals	8	1.7e-7	2.1e-08		
(C) per unit length (µmol min^{-1} cm^{-1})					
	Df	Sum Sq	Mean Sq	F value	Pr(>F)
Species	1	2.4e-5	2.4e-5	36.333	***0.000313***
Treatment	1	2.6e-7	2.6e-7	0.404	0.542844
Species:Treatment	1	7.8e-8	7.8e-8	0.120	0.737898
Residuals	8	5.2e-6	6.5e-7		

Table S3. Summary statistics for the two-way ANOVAs on the bulk oxygen consumption measurements of *A. wollastoni* and *Stichopathes* sp., with the three normalization method tested. Source data underlying table can be obtained from Supplementary Data.

2. Supplementary Methods

Coral maintenance

Two light fluorescent tubes (T8 10.000K, Power Luw Pro, Spain) with ocean blue Lee filter (LEE filters, UK) were placed above the aquarium and photoperiod was adjusted based on local light conditions (Light intensity: ~50 µmol photon m^{-2} s^{-1}). Seawater circuit was open, with the aquarium connected to a large header tank (300 L) that received the seawater pumped from the ocean. A mechanical and biological filter (Biological Filter, Aqua Medic, Germany) and a skimmer (AQUA OCEAN PRO SKP900, Spain) were used to ensure seawater filtration in the system. Seawater temperature was controlled using a chiller (HC-2200BH, Hailea Group Co., Ltd) connected to the header tank, from where the seawater was pumped into the aquarium, with adjustable flow rate, and to which it returned by overflow. The aquarium was equipped with two circulation pumps (Smart Wave Maker MOW3, Jebao Co., Ltd) and adjustable air flow. Fragments were fed twice a day from a production in the Institute, in the morning and at dawn, with a mix composed of enriched live rotifers and freshly hatched and enriched *Artemia*. The seawater inlet from the header tank was closed for at least 30 min during feeding, to ensure high food concentration and food capture optimization. All fragments survived the transport and the maintenance in *ex situ* conditions, with no necrosis observed at any time throughout the experimentation period.

Non-dimensional analysis

The transport–reaction equation describing oxygen dynamics in the coral boundary layer is given by:

$$\frac{\partial c}{\partial t} = D\nabla^2 C + \boldsymbol{u}\nabla C - R \quad (Eq.\ 1)$$

where C is the oxygen concentration, ∇ is the gradient operator, D is the molecular diffusion coefficient of oxygen at a specific temperature and salinity, in our case 1.90 x 10^{-5} cm^{-2} s^{-1} (salinity 37, 19°C), $\boldsymbol{u}$ is the velocity vector, t is the time variable, and R represents the metabolic oxygen consumption rate. To non-dimensionalize the governing equation, it is essential to identify the characteristic timescales that control the system dynamics, in particular the reaction timescale associated with metabolic oxygen consumption, diffusive transport timescale and, the advective transport timescale. These competing timescales determine the relative importance of transport and reaction processes and form the basis for defining the relevant dimensionless numbers.

Metabolic oxygen consumption was estimated from volumetric respiration rates normalized to tissue biovolume. Rates ranged from 10.0 mmol O_2 L^{-1} h^{-1} for *Stichopathes* sp. to 7.6 mmol O_2 L^{-1} h^{-1} for *A. wollastoni*. From these rates, a biological consumption timescale ($T_{reaction}$) was calculated by dividing the oxygen saturation concentration (237.5 µmol L^{-1}) by the metabolic oxygen consumption rate ($T_{reaction}$=C_0/R), effectively estimating how long it would take for the coral to deplete all available oxygen in the absence of replenishment. This resulted in characteristic timescales of ~1.2 min for *Stichopathes* sp. and ~1.8 min for *A. wollastoni*.

The transport timescale ($T_{transport}$) was estimated from the time molecular diffusion requires to cross the coral boundary layer:

$$T_{transport} = \frac{L^2}{D} \; (Eq.\ 2)$$

where L is the effective coral boundary layer thickness and D the molecular diffusion coefficient of oxygen in seawater. Since the boundary layer is ~3.4x thicker in *Stichopathes* sp., diffusive transport timescale were correspondingly longer (~27.6 min) compared to *A. wollastoni* (~2.4 min).

When using the introduced characteristic length and time scales together with a characteristic ciliary flow velocity (U), the dimensionless variables are defined as x* = x / L, t* = t D / L^2, c* = C / C0, and u* = u / U. Substituting in (*Eq. 1*) yields:

$$\frac{\partial c}{\partial t} = Pe\, D\nabla^2 C + \boldsymbol{u}\nabla C - Da \quad \textit{(Eq. 3)}$$

with Da being the Damköhler Number:

$$Da = \frac{T_{reaction}}{T_{transport}} = \frac{C_0 \cdot D}{R \cdot L^2} \textit{ (Eq. 4)}$$

When Da = 1, diffusion readily meets metabolic demand, maintaining near-ambient concentrations. When Da ≪ 1, consumption exceeds resupply, leading to steep internal gradients or even local anoxia. When Da > 1, resupply is rapid relative to consumption and oxygen supply sufficient to meet metabolic demand. While well established in analytical chemistry, the Damköhler number is not yet routinely applied in biological studies, but is attracting increasing attention (1–3). *Pe* is the Péclet number:

$$Pe = \frac{UL}{D} \textit{ (Eq. 5)}$$

This formulation represents the classical advective-diffusive Péclet number and expresses the ratio of advective to diffusive transport rates over a given spatial scale.

An additional important dimensionless number commonly used to quantify mass transfer is the Sherwood number (Sh), which represents the ratio of advective transport (here the flux with ciliary flows) to molecular diffusive transport of oxygen:

$$Sh = \frac{J_{adv}}{J_{diff}} \textit{ (Eq. 6)}$$

Together, the Péclet and Sherwood numbers provide complementary perspectives on transport processes. The Péclet number characterizes the relative importance of advection over diffusion

(4,5), and the Sherwood number quantifies how these processes enhance mass transfer across the boundary layer (6,7).

Oxygen profiles were used to determine oxygen fluxes across the coral boundary layer, with active and arrested cilia. Diffusive flux in the absence of ciliary flow is straightforward to quantify. When ciliary flows are active, however, we assume that diffusion primarily governs the flux into the vortex region, while the subsequent redistribution is controlled by recirculating flow within this quasi-closed structure. This approach allows us to approximate the net flux by separating diffusive supply from advective redistribution. They were independently analyzed to determine the limits between the lower coral boundary layer, the upper coral boundary layer, and the bulk seawater (i.e. constant part of the profile). First, the limits of the upper CBL was determined based on the linear portion of the profile, typically located above the vortices (>250 µm above tissue surface when cilia are active). This region is assumed to be governed primarily by molecular diffusion. Its upper limit was determined by the intercept between the linear concentration gradient and the constant part of the O_2 profile (background flow O_2 concentration) (8,9). Its lower limit (and the upper limit of the lower CBL) corresponded to the lower oxygen concentration value on the linear regression. In this region, diffusive flux was calculated using:

$$J_{diff} = -D \frac{\partial C}{\partial z} \text{ (Eq. 7)}$$

$\frac{\partial C}{\partial z}$ is the vertical oxygen concentration gradient, calculated by linear regression, where C is the concentration and z is the vertical distance. Importantly, to estimate the flux through the coral boundary layer (ciliary flow) it is important to determine the oxygen gradient above the vortex (5).

To calculate the advective flux induced by the coral we used:

$$J_{adv} = u\, C \text{ (Eq. 8)}$$

where u is the velocity magnitude and C is the oxygen concentration. Advective flux was averaged over the lower coral boundary layer that tended to vary in thickness due to the different profile shapes across locations and species (Figure S4). To verify the potential effect of this approach, we also averaged the advective flux over a defined and constant lower coral boundary layer thickness of 300 µm, corresponding to the height of the vortices. This approach revealed identical statistical differences, further supporting our results (Table S2).

Scanning Electron Microscopy (SEM)

Fragments of ~1 cm of both species were fixed with 2% (vol/vol) glutaraldehyde in filtered seawater. Samples were transported in this media and stored at 4°C until further processing. Samples were washed three times for 15 min in PBS 1X and then three times in milliQ water. Samples were then dehydrated in increasing ethanol concentration (30%, 50%, 70%, 80%, 90%, 96%) and dried using a Critical Point Dryer (Leica EM CPD 300). Dehydrated samples were placed on an aluminum stub and imaged using an ESEM Quanta 250 FEG (FEI, the Netherlands) at high vacuum conditions and an acceleration voltage of 2 kV.

Oxygen-sensitive nanoparticles (sensPIV)

Optical nanosensors were prepared via flash nanoprecipitation using a confined impinging jet mixer (CIJM). A polymer solution was prepared by dissolving 4.5 mg of platinum(II) meso-tetrakis(pentafluorophenyl)porphyrin (PtTFPP), 4.5 mg of Macrolex Fluorescent Yellow 10GN (MY), and 150 mg of polystyrene-co-maleic anhydride (PSMA, Mw = 250,000 g mol^{-1}) in 30 g of tetrahydrofuran (THF). Milli-Q water was used as the antisolvent. The polymer solution and antisolvent were injected into the CIJM at equal flow rates, and the resulting mixture was immediately directed into a reservoir containing additional antisolvent. The nanoparticle suspension was subsequently purged with compressed air for 3 hours to evaporate

residual THF, yielding a final concentration of 0.6 mg PSMA mL^{-1}. The resulting dispersion consisted of monodisperse nanoparticles with an average hydrodynamic diameter of 201 nm and a polydispersity index (PDI) of 0.09.

Data Processing

Image stacks were processed using ImageJ (10) and the PIVlab toolbox for MATLAB (11). Velocity fields around entire coral fragment were obtained using PIVlab, with an interrogation area of 64 pixels and 50% overlap. To quantify cilia-induced flow at the tissue surface, *Nannochloropsis* sp. microalgae were added (see above) in the chamber of the microscope slide, to act as passive tracers. Particle Tracking Velocimetry (PTV) was performed using the TrackMate plugin in ImageJ. Particles were detected using either the Thresholding Detector (intensity threshold: 50) or the Difference of Gaussians (DoG) Detector (estimated particle diameter: 5 µm; quality threshold: 1). Flow within the gastrovascular cavities was similarly analyzed by tracking particles confined within these structures. For enhanced visualization, background subtraction was first applied in ImageJ, followed by image inversion. A maximum intensity projection was then generated, producing a single image where each pixel value corresponds to the maximum intensity observed across the entire image stack. This projection was combined with the average intensity projection by assigning the maximum projection to the red channel, creating composite images for clearer visualization of flow dynamics. Finally, ciliary vortices were visualized by computing the maximum intensity projection from image stacks using ImageJ.

References

1. Jalaluddin FM, Ahmerkamp S, Marchant HK, Meyer V, Koren K, Kuypers MMM. Microenvironments on individual sand grains enhance nitrogen loss in coastal sediments. Sci Rep. (2025);15(1):16384.

2. Azizian M, Grant SB, Kessler AJ, Cook PLM, Rippy MA, Stewardson MJ. Bedforms as Biocatalytic Filters: A Pumping and Streamline Segregation Model for Nitrate Removal in Permeable Sediments. Environ Sci Technol. (2015);49(18):10993–1002.

3. Ahmerkamp S, Winter C, Janssen F, Kuypers MMM, Holtappels M. The impact of bedform migration on benthic oxygen fluxes. J Geophys Res Biogeosciences. (2015);120(11):2229–42.

4. Huysmans M, Dassargues A. Review of the use of Péclet numbers to determine the relative importance of advection and diffusion in low permeability environments. Hydrogeol J. (2005);13(5–6):895–904.

5. Shapiro OH, Fernandez VI, Garren M, Guasto JS, Debaillon-Vesque FP, Kramarsky-Winter E, et al. Vortical ciliary flows actively enhance mass transport in reef corals. Proc Natl Acad Sci. (2014);111(37):13391–6.

6. Brodkey R, Hershey H. Transport phenomena: a unified approach. Brodkey Publishing; 2003.

7. Kiørboe T. Formation and fate of marine snow: small-scale processes with large- scale implications. Sci Mar. (2001);65(S2):57–71.

8. Pacherres CO, Ahmerkamp S, Schmidt-Grieb GM, Holtappels M, Richter C. Ciliary vortex flows and oxygen dynamics in the coral boundary layer. Sci Rep. (2020);10(1):7541.

9. Revsbech NP, Jørgensen BB. Microelectrodes: their use in microbial ecology. In: Marshall KC, editor. Advances in Microbial Ecology. Boston, MA: Springer US; 1986. p. 293–352.

10. Schneider CA, Rasband WS, Eliceiri KW. NIH Image to ImageJ: 25 years of image analysis. Nat Methods. (2012);9(7):671–5.

11. Thielicke W, Sonntag R. Particle Image Velocimetry for MATLAB: Accuracy and enhanced algorithms in PIVlab. J Open Res Softw. (2021);9(1):12.
