# Supplementary material for: Ciliary flow and morphology shape mass transport at the surface and within gastrovascular cavities of black corals
Source: Commun Biol. 2026 Jun 30;9:876. doi: 10.1038/s42003-026-10531-2 (PMC13319206; doi:10.1038/s42003-026-10531-2)
Supplement: Supplementary file 3 — Description of Additional Supplementary files [file 42003_2026_10531_MOESM3_ESM.pdf]

Description of Additional Supplementary files

File name: Supplementary Data

Description: The authors declare that all data supporting the findings of this study are available within the paper in the Supplementary Data.

File name: Movie S1.

Description: Ciliary stroke dynamics.

File name: Movie S2.

Description: Acceleration of particles near the ciliated surface of A. wollastoni, using particle tracking velocimetry.

File name: Movie S3.

Description: Particles ejected from the mouth opening of Stichopathes sp.

File name: Movie S4.

Description: Flow velocity in ciliary vortices of Stichopathes sp., using particle tracking velocimetry.

File name: Movie S5.

Description: Particles traversing the dense cilia layer.

File name: Movie S6.

Description: Stichopathes sp. feeding on Artemia: (1) Artemia capture by nematocysts, mouth reorientation; (2) Artemia movement towards mouth, by ciliary flow; (3) Mouth opening, Artemia ingestion; (4) Polyp relaxation

File name: Movie S7.

Description: Internal ciliary flow and bidirectional particle displacement in gastrovascular cavities of A. wollastoni.

File name: Movie S8.

Description: Internal and external ciliary flow in tentacles of A. wollastoni.

File name: Movie S9.

Description: Internal ciliary flow in tentacles of Stichopathes sp.

File name: Movie S10.

Description: Large detrital particle in tentacle of A. wollastoni.
