# Supplementary material for: Ciliary flow and morphology shape mass transport at the surface and within gastrovascular cavities of black corals
Source: Commun Biol. 2026 Jun 30;9:876. doi: 10.1038/s42003-026-10531-2 (PMC13319206; doi:10.1038/s42003-026-10531-2)
Supplement: Supplementary file 15 — Reporting Summary [file 42003_2026_10531_MOESM15_ESM.pdf]

nature portfolio

Corresponding author(s): Mathilde Godefroid and Soeren Ahmerkamp

Last updated by author(s): May 25 , 2026

Reporting Summary

Nature Portfolio wishes to improve the reproducibility of the work that we publish. This form provides structure for consistency and transparency in reporting. For further information on Nature Portfolio policies, see our Editorial Policies and the Editorial Policy Checklist.

Statistics

For all statistical analyses, confirm that the following items are present in the figure legend, table legend, main text, or Methods section.

n/a	Confirmed	
☐	☒	The exact sample size (n) for each experimental group/condition, given as a discrete number and unit of measurement
☐	☒	A statement on whether measurements were taken from distinct samples or whether the same sample was measured repeatedly
☐	☒	The statistical test(s) used AND whether they are one- or two-sided<br>*Only common tests should be described solely by name; describe more complex techniques in the Methods section.*
☐	☒	A description of all covariates tested
☐	☒	A description of any assumptions or corrections, such as tests of normality and adjustment for multiple comparisons
☐	☒	A full description of the statistical parameters including central tendency (e.g. means) or other basic estimates (e.g. regression coefficient) AND variation (e.g. standard deviation) or associated estimates of uncertainty (e.g. confidence intervals)
☐	☒	For null hypothesis testing, the test statistic (e.g. F, t, r) with confidence intervals, effect sizes, degrees of freedom and P value noted<br>*Give P values as exact values whenever suitable.*
☐	☒	For Bayesian analysis, information on the choice of priors and Markov chain Monte Carlo settings
☐	☒	For hierarchical and complex designs, identification of the appropriate level for tests and full reporting of outcomes
☐	☒	Estimates of effect sizes (e.g. Cohen's d, Pearson's r), indicating how they were calculated

Our web collection on statistics for biologists contains articles on many of the points above.

Software and code

Policy information about availability of computer code

Data collection	sensPIV image analysis were performed with a custom-built MATLAB code. MATLAB version used was MATLAB R 2025 A.<br>PIV image capture were performed with the software FlyCapture2 (v2.13.3.61). Image analysis was performed using the MATLAB toolbox PIVlab (v3.12).<br>Particle Tracking was performed with the plugin TrackMate on Fiji (v1.54g).<br>Microsensor profiles were recorded using the software Profix from PyroScience (version unknown).
Data analysis	All statistical analysis were performed with the software R version 4.4.0.

For manuscripts utilizing custom algorithms or software that are central to the research but not yet described in published literature, software must be made available to editors and reviewers. We strongly encourage code deposition in a community repository (e.g. GitHub). See the Nature Portfolio guidelines for submitting code & software for further information.

Data

Policy information about availability of data

All manuscripts must include a data availability statement. This statement should provide the following information, where applicable:

- Accession codes, unique identifiers, or web links for publicly available datasets
- A description of any restrictions on data availability
- For clinical datasets or third party data, please ensure that the statement adheres to our policy

All data needed to evaluate the conclusions in the paper are provided in the paper, in the Supplementary Information and in the Supplementary Data files.

Research involving human participants, their data, or biological material

Policy information about studies with human participants or human data. See also policy information about sex, gender (identity/presentation), and sexual orientation and race, ethnicity and racism.

Reporting on sex and gender	NA
Reporting on race, ethnicity, or other socially relevant groupings	NA
Population characteristics	NA
Recruitment	NA
Ethics oversight	NA

Note that full information on the approval of the study protocol must also be provided in the manuscript.

Field-specific reporting

Please select the one below that is the best fit for your research. If you are not sure, read the appropriate sections before making your selection.

☐ Life sciences ☐ Behavioural & social sciences ☒ Ecological, evolutionary & environmental sciences

For a reference copy of the document with all sections, see nature.com/documents/nr-reporting-summary-flat.pdf

Ecological, evolutionary & environmental sciences study design

All studies must disclose on these points even when the disclosure is negative.

Study description	This study broadly compares the mass transfer capacity and role of ciliary flow in two black coral species with distinct morphologies.<br>For the quantitative results with statistical analysis:<br>- The effect of ciliary flow (active/inactive) on diffusive and advective fluxes were tested on both species and at 2 locations (in mouth, next to mouth) using t-tests. At each location, between 2 and 3 profiles were measured.<br>- The effect of sodium orthovanadate addition on coral metabolism was tested with a two-way ANOVA, with main effects (species and treatments) and interaction effect. For Antipathella wollastoni, 2 fragments were tested in the control (without sodium orthovanadate) and 2 others in the treatment (with sodium orthovanadate). For Stichopathes sp., 4 different fragments were tested in each treatment.
Research sample	All fragments of Antipathella wollastoni: collected at approx. 35m depth (28°01'56.0"N 15°22'32.0"W)<br>All fragments of Stichopathes sp.: collected at approx. 72m depth (28°02'23.3"N 15°21'59.2"W)<br>The samples collected are meant to represent the local population.
Sampling strategy	Fourteen 10cm-long fragments were collected for Antipathella wollastoni and for Stichopathes sp. This allows to sample a small fragment from the colony, strongly limiting our impact on the local population. They were collected while scuba diving, in ziplock bags with seawater from the collection site, then placed in cooling boxes on the boat and finally in aquariums once in the lab. The whole procedure lasted maximum 1 hour. In the laboratory, fragments were cut to obtain two ~5 cm-high fragments per colony, individually tagged, attached using underwater epoxy resin. These two fragments per colony were used independently for different test, to avoid stressing the fragments.<br>Before sampling, no sample size test was performed, but all necessary measurements were written down and the number of samples was collected accordingly.
Data collection	All data were collected by Mathilde Godefroid and Soeren Ahmerkamp and notes were taken in a handwritten lab notebook. New image files and, in general, all files generated (i.e. Excel files from the microsensor profiles) were backed up on a daily basis.
Timing and spatial scale	All A. wollastoni fragments used in the study were sampled by scuba diving on April 1st, 2025. All Stichopathes sp. fragments were

Timing and spatial scale	sampled by technical scuba diving (rebreathers) on April 7th, 2025. Stichopathes sp. was sampled 7 days later than A. wollastoni because of the conditions at sea that needed to be very good to allow sampling at 72m depth with rebreathers. All fragments per species were collected randomly within an area of approx. 30m x 30m (900m2).
Data exclusions	No data were excluded from the analyses.
Reproducibility	Since our measurements allow to understand mechanistic processes, in part through the use of visualization techniques (PIV, sensPIV), strong replication are not always necessary. For example, we repeated the sensPIV visualisations on 3 fragments per species, but conclusions drawn from these observations (showed in Fig. S3) did not change across replicates, so we did not get more images.
Randomization	Fragments were randomly sampled in the local population.
Blinding	Data collection and analysis were not performed blind to experimental conditions, due to the nature of the tests performed.

Did the study involve field work? ☒ Yes ☐ No

Field work, collection and transport

Field conditions	Samples were collected using scuba diving from the boat, seawater temperature was 19°C and outside conditions were good.
Location	A. wollastoni fragments were collected at 35m depth (28°01'56.0"N 15°22'32.0"W) and Stichopathes sp. fragments at 72m depth (28°02'23.3"N 15°21'59.2"W).
Access & import/export	All permits required for the collection of the materials used in this study were obtained: permit number SGBTM/BDM/AUTSPP/17/2024 issued from the "Ministerio para la transicion ecologica y el reto demografico" on the 19/04/2024.
Disturbance	Short fragments (max. 10 cm-long) were subsamples from colonies directly in scuba diving, to minimize the impact on the local population.

Reporting for specific materials, systems and methods

We require information from authors about some types of materials, experimental systems and methods used in many studies. Here, indicate whether each material, system or method listed is relevant to your study. If you are not sure if a list item applies to your research, read the appropriate section before selecting a response.

Materials & experimental systems

n/a	Involved in the study
☒	☐ Antibodies
☒	☐ Eukaryotic cell lines
☒	☐ Palaeontology and archaeology
☐	☒ Animals and other organisms
☒	☐ Clinical data
☒	☐ Dual use research of concern
☒	☐ Plants

Methods

n/a	Involved in the study
☒	☐ ChIP-seq
☒	☐ Flow cytometry
☒	☐ MRI-based neuroimaging

Animals and other research organisms

Policy information about studies involving animals; ARRIVE guidelines recommended for reporting animal research, and Sex and Gender in Research

Laboratory animals	The study did not involve laboratory animals
Wild animals	Coral fragments were cut from mother colonies in the field. After experimentations, all fragments were still alive and kept in aquaria at the lab facilities for a follow-up pilot experiment.
Reporting on sex	Sex was not identified because in black corals, it requires to make histological cross sections of the polyps to observe the gametes, which was not feasible in the timescale of this study, nor a direct study objective.
Field-collected samples	Fragments were placed in an open-circuit aquarium (80 L, salinity 36.8‰) at 19°C, corresponding to the temperature at both collection sites. Maintenance in aquaria was conducted according to the protocol successfully used in earlier studies. Two light fluorescent tubes (T8 10.000K, Power Luw Pro, Spain) with ocean blue Lee filter (LEE filters, UK) were placed above the aquarium and photoperiod was adjusted based on local light conditions (Light intensity: ~50 µmol photon m-2 s-1). Seawater circuit was open, with the aquarium connected to a large header tank (300 L) that received the seawater pumped from the ocean. A mechanical and

	biological filter (Biological Filter, Aqua Medic, Germany) and a skimmer (AQUA OCEAN PRO SKP900, Spain) were used to ensure seawater filtration in the system. Seawater temperature was controlled using a chiller (HC-2200BH, Hailea Group Co., Ltd) connected to the header tank, from where the seawater was pumped into the aquarium, with adjustable flow rate, and to which it returned by overflow. The aquarium was equipped with two circulation pumps (Smart Wave Maker MOW3, Jebao Co., Ltd) and adjustable air flow. Fragments were fed twice a day from a production in the Institute, in the morning and at dawn, with a mix composed of enriched live rotifers and freshly hatched and enriched Artemia. The seawater inlet from the header tank was closed for at least 30 min during feeding, to ensure high food concentration and food capture optimization.
Ethics oversight	No ethical approval or guidance was required when working on antipatharians.

Note that full information on the approval of the study protocol must also be provided in the manuscript.

Plants

Seed stocks	NA
Novel plant genotypes	NA
Authentication	NA
