# Supplementary material for: Ciliary flow and morphology shape mass transport at the surface and within gastrovascular cavities of black corals
Source: Commun Biol. 2026 Jun 30;9:876. doi: 10.1038/s42003-026-10531-2 (PMC13319206; doi:10.1038/s42003-026-10531-2)
Supplement: Supplementary file 1 — Transparent Peer Review file [file 42003_2026_10531_MOESM1_ESM.pdf]

nature portfolio

Peer Review File

Ciliary flow and morphology shape mass transport at the surface and within gastrovascular cavities of black corals

Corresponding Author: Dr Mathilde Godefroid

This file contains all reviewer reports in order by version, followed by all author rebuttals in order by version.

Version 0:

Reviewer comments:

Reviewer #1

(Remarks to the Author)
This manuscript present a in depth study on the role of cilia in determining flow conditions on the surface of black corals and its interaction with colony morphology and physiology. I found these study extremely interesting and well conducted. The different scales (colony, DBL and gastrovascular canals) investigated makes it a unique study. The manuscript is well written, the figures clear and visually appealing. Congrats to the author.
I have only some small comments as detailed below. My main concern is on the estimation of the diffusive fluxes using Fick's first law when the flow is clearly turbulent, and comparision with bluk oxygen consumption which as done under no flow conditions.

line 178 and Figure 2F: how did you define the maxima? The figures show much higher maximum flows than the one reported in the text.

line 214: "Ciliary flow also carried particles toward the mouth openings, likely improving213
feeding efficiency or ventilation. " is noted in the text, but Figure 3E show outward flow. Were there two patterns and were these alternating. It is true that some flow are directed towards the mouth (panel D) which could be to bring the food particle towards the mouth, and then an outward flow exactly at the mouth opening could be to eject the undigested detritus?

line 224: This recirculation area can be seen as thicker DBLs. Please refer to Figure 5 here as you mention the DBL thickness. (but it would become figure 4...) you could also keep these observation for the section on DBL, line 298-301.

Diffusive fluxes: line 302 and line 658, the Fick'ss 1st law is only valid for 2D diffusive fluxes (constant concentration on X and y, and only varying on z) in laminar flows. Although it is often assumed in experiment with corals, this is mostly done using massive shapes corals and not branching one. Here you use branching corals and show a turbulent flow (due to colony porphology and cilia beating). I am not sure that the assumption for the Fick's law are valid in this case. As it is not possible to estimate the variation in concentration, i would suggest that you discuss this limitation and note that the fluxes may have been underestimated due to these constraints and how this affect the following discussion comparing diffusive vs advection fluxes. It should not change much for A wollastoni but what about for Stichopathes that show limitation by diffusion.

Microsensor measurement: line 280, "additional oxygen microsensor profiles at the tissue surface and in the mouth opening of both coral species under flow conditions" I could not find the current velocity used in the flow chamber in the method, could you give it?

Bulk respiration measurements (line 682): During these measurements, the coral fragments were enclosed in vessel under no flow conditions (but for the regular inversion of the vials). This would cause oxygen depletion at the surface of the tissue (figure 4) and could minor "true" respiration rates as the measured one could have been limited by diffusion, no? Could that affect the comparision with the microsensor derived fluxes (but see comments above)? If the bulk respiration rates were limited by diffusion, when comparing the Da, you would be comparing to a respiration rates already limited. I am not sure I am understanding well the statement "oxygen delivery into the tissue is ultimately limited by molecular diffusion" which may be key to your assumption, do you mean that to oxygen penetration within the tissue is diffusion driven? but what about of all

the upper layer where you show that advection is important too?

Reviewer #2

(Remarks to the Author)
This manuscript investigates how colony morphology and ciliary activity shape mass transport at the coral–seawater interface and within gastrovascular cavities in two black coral species with contrasting forms, the whip-like Stichopathes sp. and the branched Antipathella wollastoni. By combining high-speed videography, SEM/light microscopy, PIV/PTV, oxygen-sensitive sensPIV, microsensor profiling, and non-dimensional analyses, the authors show that colony architecture strongly modifies local flow fields, that both species possess active epidermal cilia, and that ciliary vortices enhance near-surface transport. The study further suggests that the functional role of ciliary flow differs between the two species: in Stichopathes sp. ciliary activity appears to strongly enhance oxygen supply and net exchange, whereas in A. wollastoni it mainly redistributes solutes locally while the larger exchange surface reduces diffusion limitation. The manuscript also presents evidence that ciliary activity contributes to internal circulation within gastrovascular cavities and that skeletal spines help structure this internal transport system. Overall, the work is technically ambitious, visually compelling, and potentially important for understanding the link between form, function, and transport processes in sessile marine organisms. Overall, this is a creative and technically impressive study with clear novelty, I recommend it for publication in its present form.

Reviewer #3

(Remarks to the Author)
General Comments
This study investigates microscale fluid dynamics and oxygen transport around black coral cilia using advanced imaging techniques, specifically microscale PIV and oxygen-sensitive PIV. The application of these high-resolution microfluidic measurements to ciliary-scale biological processes is highly innovative and represents the most compelling aspect of this work. While micro-PIV is a mature tool in engineering, its integration with oxygen sensing to resolve coral microenvironments is novel and has considerable potential.
However, in its current form, the manuscript is not suitable for publication as a Letter in Communications Biology. The overall structure is loose and overly descriptive, lacking sufficient synthesis, rigorous quantification, and mechanistic depth. The authors primarily present imaging results followed by qualitative descriptions, without clearly distilling the underlying physical principles or developing a strong conceptual framework.
For a short-format, high-impact journal, the manuscript must be substantially streamlined. I recommend that the authors refocus the central scientific message and address the following major issues:
1. Physical framework: There is significant ambiguity regarding the governing flow regimes. The manuscript does not clearly distinguish whether the observed transport processes are dominated by shear-driven advection, turbulence, or purely molecular diffusion. This lack of clarity weakens the physical interpretation of the results.
2. Discussion structure: The biological and ecological implications are not clearly separated from the physical results. A more structured discussion is needed to explicitly link microscale flow processes to their biological consequences, allowing for a clearer inference of how these dynamics influence coral function and fitness.
Overall, while the measurements are interesting and potentially impactful, the manuscript requires substantial revision in both its structural organization and scientific framing.
Specific Comments:
L67–69: The definitions of the diffusive boundary layer (DBL) and molecular diffusion are conflated and require clarification. These represent distinct physical mechanisms. The authors should clearly specify the relevant transport regime at the investigated scale. At the ciliary level, is transport still influenced by turbulent shear-induced processes, or has it reached the molecular diffusion regime? Consider whether the Kolmogorov microscale is relevant for interpreting the fluid–tissue interface?
L71–74: To better position the novelty of this work, the introduction should include a more structured review of flow–organism interactions across spatial scales. Previous studies (e.g., Reidenbach et al., 2006) have focused on colony-scale (~cm) interactions, whereas this study targets ciliary-scale microflows. Explicitly highlighting this scale transition would strengthen the justification of the study's novelty.
L128–147: The description of Figure 1 is overly verbose. Instead of relying on qualitative descriptions, the authors should provide quantitative geometric metrics (e.g., surface-area-to-volume ratio, branch spacing, or fractal dimension) to enable a more rigorous comparison between the two species. Additionally, the manuscript would benefit from consolidating figures and removing redundant content, focusing only on the most essential results.
L227, L316, L327: Formal definitions and updated references are required for the Péclet (Pe), Sherwood (Sh), and Damköhler (Da) numbers. These dimensionless parameters are central to the interpretation of transport processes. Their use should be clearly grounded in established literature in the Results section, with detailed formulations and assumptions provided in the Methods.
L400–413: The claim of the "first observation of internal flow" based on high-speed videography and a single PTV dataset is not yet convincing. This section remains largely descriptive. More robust replication and/or a deeper mechanistic analysis are needed to support such a strong conclusion. It is also unclear what generalizable physical insight is gained from this observation.
L463 & Methods: The Methods section should be significantly streamlined. Redundant or highly detailed descriptions should be moved to the Supplementary Information to better align with the Letter format.
L667: The use of pure molecular diffusion coefficients is conceptually problematic. Within the DBL, transport is often still influenced by residual flow or cilia-induced mixing. The distinction between turbulent transport and molecular diffusion should be more carefully addressed, supported by updated literature from the past decade.

Version 1:

Reviewer comments:

Reviewer #1

(Remarks to the Author)
Thanks for the revised manuscript. You cleared some points I raised (outflow from the mouth, non planar diffusion and diffusion limitation in the bulk measurements) and improved the manuscript following my and reviewer 3 comments. I recommend the manuscript for publication in its present state (minus a typo line 302 "reduced on by" remove the on).

Reviewer #3

(Remarks to the Author)
In this revised version, the authors have clarified the physical definitions and flow regime, facilitating a clearer connection from coral colony-scale flows down to the microscopic cilia scale. While the diverse micro-geometries and limited case studies make the results appear more descriptive, the novelty and difficulty of capturing such intricate, multi-scale measurements are highly impressive. This work provides valuable insights that will attract a broad readership in both fluid mechanics and biology. I am pleased to recommend its publication.

Open Access This Peer Review File is licensed under a Creative Commons Attribution 4.0 International License, which permits use, sharing, adaptation, distribution and reproduction in any medium or format, as long as you give appropriate credit to the original author(s) and the source, provide a link to the Creative Commons license, and indicate if changes were made.
In cases where reviewers are anonymous, credit should be given to 'Anonymous Referee' and the source.
The images or other third party material in this Peer Review File are included in the article's Creative Commons license, unless indicated otherwise in a credit line to the material. If material is not included in the article's Creative Commons license and your intended use is not permitted by statutory regulation or exceeds the permitted use, you will need to obtain permission directly from the copyright holder.
To view a copy of this license, visit https://creativecommons.org/licenses/by/4.0/

Responses to Reviewers

Ciliary flow and morphology shape mass transport at the surface and within gastrovascular cavities of black corals

Mathilde Godefroid *et al.*

*Corresponding authors: Mathilde Godefroid, godefroid.mathilde1@gmail.com;

Soeren Ahmerkamp, soeren.ahmerkamp@io-warnemuende.de

Referee comments	Responses
**Reviewer #1 (Remarks to the Author):** This manuscript present a in depth study on the role of cilia in determining flow conditions on the surface of black corals and its interaction with colony morphology and physiology. I found these study extremely interesting and well conducted. The different scales (colony, DBL and gastrovascular canals) investigated makes it a unique study. The manuscript is well written, the figures clear and visually appealing. Congrats to the author. I have only some small comments as detailed below. My main concern is on the estimation of the diffusive fluxes using Fick's first law when the flow is clearly turbulent, and comparison with bulk oxygen consumption which was done under no flow conditions.	We would like to thank the reviewer for the positive evaluation and insightful and constructive comments.
1. line 178 and Figure 2F: how did you define the maxima? The figures show much higher maximum flows than the one reported in the text.	We did not use the absolute maximum, but instead report the 90th percentile to avoid bias from extreme values (e.g. outliers) and to better represent the dominant flow field. This was specified in the text: *"In Stichopathes sp., average near-surface velocities were 341 ± 3 µm s^{-1}, with the 90th percentile approaching 541 µm s^{-1} (Figure 2F)."* (L187-188)
2. line 214: "Ciliary flow also carried particles toward the mouth openings, likely improving feeding efficiency or ventilation." is noted in the text, but Figure 3E show outward flow. Were there two patterns and were these alternating. It is true that some flow are directed towards the mouth (panel D) which could be to bring the	We thank the reviewer for raising this interesting aspect. Ciliary flow was consistently observed to transport particles upward along the mouth walls (panel D), while outward-directed vortical flows occurred near the mouth openings (panel E). We therefore agree with the reviewer's suggestion that undigested detritus might be ejected. We clarified this behavior accordingly in the revised manuscript,

food particle towards the mouth, and then an outward flow exactly at the mouth opening could be to eject the undigested detritus?	including an additional video depicting this behavior: *"Ciliary flow was observed carrying particles toward the mouth openings, likely enhancing feeding efficiency or ventilation; in addition, particles were occasionally observed being ejected from the mouth (Movie S3), indicating that ciliary transport can also facilitate outward fluxes, such as waste removal."* (L222-224)
3. line 224: This recirculation area can be seen as thicker DBLs. Please refer to Figure 5 here as you mention the DBL thickness. (but it would become figure 4...) you could also keep these observation for the section on DBL, line 298-301.	We thank the reviewer for this comment. We realized that the sentence was redundant and removed it, as we basically said the same in L310-312.
4. Diffusive fluxes: line 302 and line 658, the Fick's 1st law is only valid for 2D diffusive fluxes (constant concentration on X and y, and only varying on z) in laminar flows. Although it is often assumed in experiment with corals, this is mostly done using massive shapes corals and not branching one. Here you use branching corals and show a turbulent flow (due to colony morphology and cilia beating). I am not sure that the assumption for the Fick's law are valid in this case.<br>As it is not possible to estimate the variation in concentration, i would suggest that you discuss this limitation and note that the fluxes may have been underestimated due to these constraints and how this affect the following discussion comparing diffusive vs advection fluxes. It should not change much for A wollastoni but what about for Stichopathes that show limitation by diffusion.	The flow in our setup was laminar (Re = 1.8 - 5.9), i.e. dominated by viscous forces. By applying Fick's first law, we assume quasi-steady conditions, even in the presence of ciliary flow, i.e. vortical flows (Movie S4). Our approach estimates the diffusive flux above the mostly closed ciliary vortex (compare Figure 6), i.e. the flux entering the vortex, which serves as a proxy for coral uptake, consistent with approaches such as Pacherres et al. 2020 (Scientific Reports). We acknowledge, however, that some temporal variability cannot be fully excluded. In addition, the branching morphology introduces potential three-dimensional effects. Approximating the coral branch as a cylindrical structure and accounting for rotational symmetry increases the estimated flux by up to a factor of ~2.2, based on the measured spatial extent of the coral boundary layer. Applying this geometric correction would raise the Damköhler number for A. wollastoni to Da=1.5, indicating that transport is not diffusion-limited, whereas for Stichopathes the corrected value remains low (Da=0.1), consistent with diffusion limitation. Importantly, this interpretation is independently supported by the observed oxygen concentration in proximity of the mouth opening. In Stichopathes, oxygen concentrations already decline ~1 mm above the mouth opening in the absence of ciliary flow. This demonstrates that diffusion limitation is present under quiescent conditions and is not an artefact of dimensionality or geometric simplification. We are therefore confident that our conclusions regarding the contrasting transport regimes are robust, and that the use of a 1D

	approximation does not alter the qualitative interpretation but rather provides a conservative estimate.<br><br>We are now acknowledging the limitations in the text:<br>- *“These net fluxes should be regarded as conservative estimates, as the three-dimensional nature of the coral morphology may introduce out-of-plane components, and additional unsteady dynamics, such as polyp movements, could further influence the net fluxes.”* (L319-321)<br>- *“We note that the estimation of the Da number relies on the respiration rates determined in closed vials. Although mixing in the vials was promoted by regular inversions, diffusion-limitation and boundary layer formation cannot be fully excluded, potentially leading to an underestimation of respiration rates and consequently slightly overestimated Da numbers.”* (L361-365)<br><br>**Reference**<br>Pacherres, C.O., Ahmerkamp, S., Schmidt-Grieb, G.M. et al. Ciliary vortex flows and oxygen dynamics in the coral boundary layer. Scientific Reports 10, 7541 (2020). https://doi.org/10.1038/s41598-020-64420-7
5. Microsensor measurement: line 280, "additional oxygen microsensor profiles at the tissue surface and in the mouth opening of both coral species under flow conditions" I could not find the current velocity used in the flow chamber in the method, could you give it?	We agree and have added that the applied flow velocity was between 0.4 and 1.0 cm s^{-1}. In response to reviewer 3 we are now also more extensively describing the flow regimes.<br>For example:<br>- *“…(free stream velocities of approx. 0.4 cm s^{-1}).”* (L293)<br>- *“Our experiments were conducted under laminar, low-flow conditions. Under turbulent background flow, however, ciliary vortices are likely to interact with ambient turbulent regimes, leading to more complex and potentially intermittent transport dynamics. Such interactions may enhance or disrupt local mixing, alter coral boundary layer thickness dynamically, and thereby modulate mass transfer rates at the tissue-fluid interface. Investigating these coupled processes under realistic turbulent conditions represents an important avenue for future work.”* (L485-490)
6. Bulk respiration measurements (line 682): During these measurements, the coral fragments were enclosed in vessel under no	We acknowledge that despite efforts to enhance ventilation through regular inversion of the vials, diffusion limitation cannot be fully excluded and may

flow conditions (but for the regular inversion of the vials). This would cause oxygen depletion at the surface of the tissue (figure 4) and could minor "true" respiration rates as the measured one could have been limited by diffusion, no? Could that affect the comparison with the microsensor derived fluxes (but see comments above)? If the bulk respiration rates were limited by diffusion, when comparing the Da, you would be comparing to a respiration rates already limited.	have led to a slight underestimation of respiration rates. However, if respiration rates were higher, the resulting Damköhler numbers (with respiration in the denominator) would decrease accordingly, implying that our numbers are conservative.<br>We have now explicitly acknowledged this limitation in the revised manuscript and clarified its implications for the comparison with microsensor-derived fluxes and for the interpretation of the Damköhler number: *"We note that the estimation of the Da number relies on the respiration rates determined in closed vials. Although mixing in the vials was promoted by regular inversions, diffusion-limitation and boundary layer formation cannot be fully excluded, potentially leading to an underestimation of respiration rates and consequently slightly overestimated Da numbers."* (L361-365)
7. L717: I am not sure I am understanding well the statement "oxygen delivery into the tissue is ultimately limited by molecular diffusion" which may be key to your assumption, do you mean that to oxygen penetration within the tissue is diffusion driven? but what about of all the upper layer where you show that advection is important too?	We agree that this statement is misleading. Since it is not necessary to support the understanding of this section, we have removed the sentence.
**<u>Reviewer #2 (Remarks to the Author):</u>**<br>This manuscript investigates how colony morphology and ciliary activity shape mass transport at the coral–seawater interface and within gastrovascular cavities in two black coral species with contrasting forms, the whip-like Stichopathes sp. and the branched Antipathella wollastoni. By combining high-speed videography, SEM/light microscopy, PIV/PTV, oxygen-sensitive sensPIV, microsensor profiling, and non-dimensional analyses, the authors show that colony architecture strongly modifies local flow fields, that both species possess active epidermal cilia, and that ciliary vortices enhance near-surface transport. The study further suggests that the functional role of ciliary flow differs between the two species: in Stichopathes sp. ciliary activity appears to strongly enhance oxygen supply and net exchange, whereas in A. wollastoni it mainly	We thank the reviewer for the positive evaluation of the manuscript.

redistributes solutes locally while the larger exchange surface reduces diffusion limitation. The manuscript also presents evidence that ciliary activity contributes to internal circulation within gastrovascular cavities and that skeletal spines help structure this internal transport system. Overall, the work is technically ambitious, visually compelling, and potentially important for understanding the link between form, function, and transport processes in sessile marine organisms. Overall, this is a creative and technically impressive study with clear novelty, I recommend it for publication in its present form.	
**<u>Reviewer #3 (Remarks to the Author):</u>** This study investigates microscale fluid dynamics and oxygen transport around black coral cilia using advanced imaging techniques, specifically microscale PIV and oxygen-sensitive PIV. The application of these high-resolution microfluidic measurements to ciliary-scale biological processes is highly innovative and represents the most compelling aspect of this work. While micro-PIV is a mature tool in engineering, its integration with oxygen sensing to resolve coral microenvironments is novel and has considerable potential.	We thank the reviewer for highlighting the innovation of our work.
However, in its current form, the manuscript is not suitable for publication as a Letter in Communications Biology. The overall structure is loose and overly descriptive, lacking sufficient synthesis, rigorous quantification, and mechanistic depth. The authors primarily present imaging results followed by qualitative descriptions, without clearly distilling the underlying physical principles or developing a strong conceptual framework. For a short-format, high-impact journal, the manuscript must be substantially streamlined. I recommend that the authors refocus the central scientific message and address the following major issues:<br><u>Physical framework</u>: There is significant ambiguity regarding the governing flow	 <br><br><br><br><br><br><br><br><br><br><br><br><br>Black corals typically inhabit depths greater than ~20 m, where ambient free-stream velocities near our

regimes. The manuscript does not clearly distinguish whether the observed transport processes are dominated by shear-driven advection, turbulence, or purely molecular diffusion. This lack of clarity weakens the physical interpretation of the results.	study site range between 0–15 cm s^{-1} (e.g. Palacios-Castillo et al. 2026). In designing our experiments, we explicitly considered the rapid decay of flow within the benthic boundary layer toward the viscous sublayer. Accordingly, we applied chamber flow velocities of ~0.4–1 cm s^{-1}, consistent with values used in previous studies on black corals (Rakka et al. 2020) and scleractinian corals (Pacherres et al. 2020; Ahmerkamp et al. 2022). Using a characteristic branch width of ~1 mm, we estimate a Reynolds number of $Re \approx 4$, clearly placing the system in the laminar regime. Under these conditions, viscous forces dominate and turbulent transport can be neglected. Mass exchange between the coral and the surrounding water is therefore governed by the interplay between ciliary advection and molecular diffusion. This regime is appropriately described by the Péclet, Sherwood, and Damköhler numbers. To avoid ambiguity, we have revised the manuscript to include a dedicated paragraph that explicitly defines the governing low-flow regime (L140-144), we introduce the relevant non-dimensional numbers with their derivation in the methods, and clarify their implications for transport processes (Supplementary Methods).<br><br>**References**<br>- Ahmerkamp, S., Jalaluddin, F. M., Cui, Y., Brumley, D. R., Pacherres, C. O., Berg, J. S., ... & Behrendt, L. (2022). Simultaneous visualization of flow fields and oxygen concentrations to unravel transport and metabolic processes in biological systems. Cell Reports Methods, 2(5).<br>- Pacherres, C. O., Ahmerkamp, S., Schmidt-Grieb, G. M., Holtappels, M., & Richter, C. (2020). Ciliary vortex flows and oxygen dynamics in the coral boundary layer. Scientific reports, 10(1), 7541.<br>- Palacios-Castillo, L., Navarro-Mayoral, S., Godefroid, M., García-Herrera, C., Tuya, F., Haroun, R., & Otero-Ferrer, F. (2026). Sex matters: female black corals experience higher stress under different current velocities. Coral Reefs, 45(1), 257-271.<br>- Rakka, M., Orejas, C., Maier, S. R., Van Oevelen, D., Godinho, A., Bilan, M., & Carreiro-Silva, M. (2020). Feeding biology of a habitat-forming antipatharian in the Azores Archipelago. Coral Reefs, 39(5), 1469-1482.
<u>Discussion structure</u>: The biological and ecological implications are not clearly separated from the physical results. A more structured discussion is needed to explicitly link	In black corals, and as highlighted in our study, ecological implications are tightly coupled to the underlying physical processes, making a strict separation between the two inherently challenging.

microscale flow processes to their biological consequences, allowing for a clearer inference of how these dynamics influence coral function and fitness.<br>Overall, while the measurements are interesting and potentially impactful, the manuscript requires substantial revision in both its structural organization and scientific framing.	Nevertheless, we appreciate the reviewer's comment and have strengthened the physical framing of the manuscript accordingly. Specifically, we have clarified the assumptions and limitations of our approach, restructured parts of the manuscript to improve clarity (Introduction and Discussion sections), provided a more explicit description of the flow regime and also integrated the importance of morphology in turbulent flow. In addition, we now derive the relevant non-dimensional numbers and include a more detailed description of these numbers in the Supplementary Methods.
8. L67–69: The definitions of the diffusive boundary layer (DBL) and molecular diffusion are conflated and require clarification. These represent distinct physical mechanisms.	We agree that our wording blended two related but distinct concepts (diffusive boundary layer as a spatial region and molecular diffusion as a transport process). We have revised the introduction section and this section was also adjusted: "*Here, mass-transfer is dominated by molecular diffusion and the slow transport leads to the formation of a diffusive boundary layer (DBL), which thickness governs the exchange of oxygen, nutrients and waste products (20). For corals, the DBL thickness, operationally defined as the point where solute concentrations reach ~99%, depends on ambient flow (12,21–23), is influenced by surface topography and polyp behavior, creating coral-specific boundary layer, hereafter referred to as "coral boundary layer" (CBL) (24–29).*" (L79-85)
9. The authors should clearly specify the relevant transport regime at the investigated scale. At the ciliary level, is transport still influenced by turbulent shear-induced processes, or has it reached the molecular diffusion regime? Consider whether the Kolmogorov microscale is relevant for interpreting the fluid–tissue interface?	Based on a characteristic flow velocity of ~0.4 - 1 cm s^{-1} and a branch width of ~1 mm, we estimate a Reynolds number of $Re \approx 4$, clearly indicating laminar flow and excluding turbulent transport. At the scale of the ciliary boundary layer, viscous forces dominate and transport is governed by molecular diffusion, or by ciliary-driven advection when active. Turbulent processes are therefore not relevant at this scale, and the Kolmogorov microscale does not constrain transport at the fluid-tissue interface in our system. We have clarified this in the revised manuscript by explicitly outlining the governing transport regime and relevant non-dimensional numbers and we rewrote the introduction.<br><br>For example: "*As flow approaches the tissue surface, it is progressively attenuated, forming a thin flow boundary layer. Within this sub-millimeter region, below the Kolmogorov microscale ($\eta \approx 10^{-3}$ m in seawater; e.g. 19),*

	turbulence is dissipated and t viscous forces dominate. Here, mass-transfer is dominated by molecular diffusion and the slow transport leads to the formation of a diffusive boundary layer (DBL), which thickness governs the exchange of oxygen, nutrients and waste products (20).” (L76-81)
10. L71–74: To better position the novelty of this work, the introduction should include a more structured review of flow–organism interactions across spatial scales. Previous studies (e.g., Reidenbach et al., 2006) have focused on colony-scale (~cm) interactions, whereas this study targets ciliary-scale microflows. Explicitly highlighting this scale transition would strengthen the justification of the study’s novelty.	We agree that the scale transition from organism/colony to cilia is rather implicit in the present form. We have re-written the introduction to put more emphasis on the different scales and clearly place our study into the submilimeter-milimeter scale. We are further acknowledging the previous works on the importance of flow-morphology interactions in turbulent flow. See changes in L74-86.
11. L128–147: The description of Figure 1 is overly verbose. Instead of relying on qualitative descriptions, the authors should provide quantitative geometric metrics (e.g., surface-area-to-volume ratio, branch spacing, or fractal dimension) to enable a more rigorous comparison between the two species. Additionally, the manuscript would benefit from consolidating figures and removing redundant content, focusing only on the most essential results.	We agree that quantitative descriptors are important to compare morphologies, and we now additionally added the branch/stem diameter, branching density and inter-branch spacing in Figure S1A. Only the fractal dimension was not calculated, as this would require a full three-dimensional reconstruction of the morphology across all investigated scales.<br>We condensed the figure caption of Figure 1, while still retaining some descriptive component, as we believe it is important to introduce the morphology of the studied species, in particular for antipatharians, which are not well-known organisms even among coral biologists. For example, the presence of polyps only on one side of the stem is an important trait to consider when interpreting the physiological results later in the manuscript. We also referenced Figure S1 in the figure caption.
12. L227, L316, L327: Formal definitions and updated references are required for the Péclet (Pe), Sherwood (Sh), and Damköhler (Da) numbers. These dimensionless parameters are central to the interpretation of transport processes. Their use should be clearly grounded in established literature in the Results section, with detailed formulations and assumptions provided in the Methods.	We have now formally derived the relevant non-dimensional numbers from the scalar transport equations within the Supplementary Methods section and have included references to prior studies where these numbers have been applied to describe the close coupling between biological processes and physical transport phenomena.<br><br>For example: “*To evaluate this, we compared the characteristic timescales of oxygen consumption and transport using the Damköhler number (Da), a dimensionless parameter expressing the balance between*

	metabolic demand and mass-transfer supply (for Da calculation and derivation, see Supplementary Methods, Eq. 1 - 4, (30,68–70))." (L343-347).
13. L400–413: The claim of the "first observation of internal flow" based on high-speed videography and a single PTV dataset is not yet convincing. This section remains largely descriptive. More robust replication and/or a deeper mechanistic analysis are needed to support such a strong conclusion. It is also unclear what generalizable physical insight is gained from this observation.	We thank the reviewer for this important point and agree that the original statement was too strong. We have revised the wording accordingly. We note that extensive replication is inherently challenging for mesophotic specimens (~70 m depth, sampled with technical divers). While only one dataset allowed quantitative velocity extraction via PTV due to specimen motion, multiple independent high-speed recordings consistently show systematic, bidirectional particle transport (Movie S7, additional videos now provided as Movies S8 and S9). We therefore use the qualitative reproducibility across independent observations (different species+replicates and various locations) as supporting evidence for the presence of internal flow. We agree that a full mechanistic analysis is beyond the scope of the current manuscript. However, we argue that documenting this previously unresolved internal transport process provides a relevant physical insight into black coral mass transfer at the tissue-cavity interface which links well to the fluid-tissue interfaces. To better reflect this, we have reframed the result and explicitly highlight its implications as a testable hypothesis for future work.<br><br>*"These results represent a first direct observation of internal flow in hexacorals using naturally occurring particles in combination with natural ingestion (probably induced by ciliary flows), extending previous indirect inferences based on particles or dye injections through the oral opening of scleractinian corals (52,53,71)."* (L437-440)
14. L463 & Methods: The Methods section should be significantly streamlined. Redundant or highly detailed descriptions should be moved to the Supplementary Information to better align with the Letter format.	We thank the reviewer for this helpful suggestion and agree that the Methods section was overly detailed and partially repetitive. We have therefore substantially condensed the Methods in the main text and moved more detailed descriptions to the Supplementary Method sections.
15. L667: The use of pure molecular diffusion coefficients is conceptually problematic. Within the DBL, transport is often still influenced by residual flow or cilia-induced	We agree that the distinction between molecular diffusion and flow-driven transport within the coral boundary layer requires careful consideration. In our study, we explicitly focus on the interplay between

mixing. The distinction between turbulent transport and molecular diffusion should be more carefully addressed, supported by updated literature from the past decade.	molecular diffusion and advection induced by ciliary flows within the coral boundary layer. In cases where active ciliary motion is present, transport is therefore not treated as purely diffusive but instead reflects this coupled advective–diffusive regime. Only in the absence of detectable ciliary activity we approximate transport using molecular diffusion coefficients. Importantly, the flow conditions in our experiments are characterized by low Reynolds numbers, indicating a laminar flow regime in which turbulent transport can be neglected. Under these stationary conditions, and given the small spatial scales considered, we do not expect a direct contribution from residual flows or turbulent mixing. We have clarified this distinction in the revised manuscript (e.g., L76-79) and expanded the introduction and discussion to better reflect the relevant transport regimes, spatial scales and integrate recent literature (e.g., L74-80, L485-490). We have further discussed the limitation of our flux estimations, based on suggestions from reviewer 1 (e.g., L361-365). We also acknowledge that it will be important for future work to investigate how ciliary flows interact with turbulence and how these interactions modify the surrounding flow field and mass-transfer (L489-490).
